# AHRR and SFRP2 in primary versus recurrent high-grade serous ovarian carcinoma and their prognostic implication

**DOI:** 10.1038/s41416-023-02550-1

**Published:** 2024-02-09

**Authors:** Nanna Monjé, Mihnea P. Dragomir, Bruno V. Sinn, Inga Hoffmann, Anuar Makhmut, Tincy Simon, Catarina A. Kunze, Jana Ihlow, Wolfgang D. Schmitt, Jonathan Pohl, Iris Piwonski, Sofya Marchenko, Carlotta Keunecke, Teodor G. Calina, Francesca Tiso, Hagen Kulbe, Caroline Kreuzinger, Dan Cacsire Castillo-Tong, Jalid Sehouli, Elena I. Braicu, Carsten Denkert, Silvia Darb-Esfahani, Kirsten Kübler, David Capper, Fabian Coscia, Markus Morkel, David Horst, Christine Sers, Eliane T. Taube

**Affiliations:** 1 Charité—Universitätsmedizin Berlin, Corporate Member of Freie Universität Berlin and Humboldt-Universität zu Berlin, Institute of Pathology, Charitéplatz 1, 10117 Berlin, Germany; 2 German Cancer Consortium (DKTK), Partner Site Berlin, and German Cancer Research Center (DKFZ), Heidelberg, Germany; 3 Berlin Institute of Health (BIH), Berlin, Germany; 4 Max-Delbrück-Center for Molecular Medicine in the Helmholtz Association (MDC), Spatial Proteomics Group, Berlin, Germany; 5 Charité—Universitätsmedizin Berlin, corporate member of Freie Universität Berlin and Humboldt-Universität zu Berlin, Department for Gynecology with the Center for Oncologic Surgery Charité Campus Virchow-Klinikum, Charitéplatz 1, 10117 Berlin, Germany; 6 TGC Ventures UG, Berlin, Germany; 7 Center of Functional Genomics, Berlin Institute of Health at Charité—Universitätsmedizin Berlin, Charitéplatz 1, 10117 Berlin, Germany; 8 Department of Hematology, Oncology and Cancer Immunology, Charité—Universitätsmedizin Berlin, corporate member of Freie Universität Berlin and Humboldt-Universität zu Berlin, Hindenburgdamm 30, 12203 Berlin, Germany; 9 Translational Gynecology Group, Department of Obstetrics and Gynecology, Comprehensive Cancer Center, Medical University of Vienna, Vienna, Austria; 10 Institute of Pathology, University Hospital Gießen and Marburg, Marburg, Germany; 11 Institute of Pathology, Berlin-Spandau, Stadtrandstraße 555, 13589 Berlin, Germany; 12 Cancer Program, Broad Institute of MIT and Harvard, Cambridge, MA USA; 13 Center for Cancer Research, Massachusetts General Hospital, Harvard Medical School Teaching Hospital, Charlestown, MA USA; 14 Department of Neuropathology, Charité—Universitätsmedizin Berlin, Corporate Member of Freie Universität Berlin, Humboldt-Universität zu Berlin, Berlin, Germany

**Keywords:** Prognostic markers, Ovarian cancer

## Abstract

**Background:**

The aim of this study was to analyse transcriptomic differences between primary and recurrent high-grade serous ovarian carcinoma (HGSOC) to identify prognostic biomarkers.

**Methods:**

We analysed 19 paired primary and recurrent HGSOC samples using targeted RNA sequencing. We selected the best candidates using in silico survival and pathway analysis and validated the biomarkers using immunohistochemistry on a cohort of 44 paired samples, an additional cohort of 504 primary HGSOCs and explored their function.

**Results:**

We identified 233 differential expressed genes. Twenty-three showed a significant prognostic value for PFS and OS in silico. Seven markers (*AHRR, COL5A2, FABP4, HMGCS2, ITGA5, SFRP2 and WNT9B*) were chosen for validation at the protein level. AHRR expression was higher in primary tumours (*p* < 0.0001) and correlated with better patient survival (*p* < 0.05). Stromal SFRP2 expression was higher in recurrent samples (*p* = 0.009) and protein expression in primary tumours was associated with worse patient survival (*p* = 0.022). In multivariate analysis, tumour AHRR and SFRP2 remained independent prognostic markers. In vitro studies supported the anti-tumorigenic role of AHRR and the oncogenic function of SFRP2.

**Conclusions:**

Our results underline the relevance of AHRR and SFRP2 proteins in aryl-hydrocarbon receptor and Wnt-signalling, respectively, and might lead to establishing them as biomarkers in HGSOC.

## Introduction

Ovarian cancer (OC) is the fifth leading cause of cancer death for women in Western countries [1]. Among several histological subtypes, high-grade-serous ovarian carcinoma (HGSOC) is the most common one, often diagnosed at a late stage when the tumour has already spread within the abdominal cavity [2]. Consequently, HGSOC accounts for the highest number of deaths among ovarian cancer patients and 5-year survival is as low as 43% [3]. A major setback for successful treatment of OC patients is relapse of primary tubo-ovarian carcinomas, which occurs after primary response to therapy and is experienced in approximately 80% of patients. The median time from primary diagnosis to recurrence is only 16 months [4]. As these recurrences often become resistant to conventional therapies, treatment options are limited and overall life expectancy for these patients is short.

Despite its immense clinical importance, data on treatment resistance mechanisms and the unique biology of relapsed HGSOC is still sparse. An explanation might be the difficulty in obtaining matched primary and recurrent samples, as surgery is not generally performed in the relapsed stage and candidates for surgical treatment must be chosen conscientiously [5].

Recent research from transnational consortia addressed these problems [6–8]. It seems that apart from the known dysregulation of homologous recombination deficiency (HRD) and *TP53*, both of which play an important role in tumour initiation of HGSOC [9, 10], additional molecular mechanisms, for example, the upregulation of a therapy resistance-related drug efflux pump (MDR1) become important for tumour preservation after primary chemotherapy [8]. Analyses on the temporal heterogeneity in immune response [6, 11, 12] and angiogenesis [13] in recurrent HGSOC concentrated on the evaluation of specific immunohistochemically (IHC) detectable markers. However, to date, there has been no exploratory analysis of differentially expressed genes in the recurrence of HGSOC that includes protein-based validation.

We aimed to discover molecules that mark the progression from primary to recurrent samples under the assumption that these markers would be more pronounced in recurrences. Therefore, we analysed signalling pathways involved in the process of tumour recurrence. We further wondered if the detected differentially expressed markers were merely an adaption to tumour progression or if their initial presentation in the primary tumour would also indicate prognostic potential and they could thus serve as biomarkers. We focused on the prognostic impact of a small subset of promising candidates in a cohort of 504 primary HGSOC samples with well-annotated survival data to assess their clinical utility for patient stratification and survival prediction. AHRR and SFRP2 were demonstrated to be consistently clinically relevant markers throughout these analyses and exploratory studies were performed to understand their upstream regulation and downstream function.

## Methods

### Patient Cohort

Three independent patient collectives were included in this study. The first one consists of paired primary and recurrent HGSOC (PRI-REC-Cohort; Screening (*n* = 19) and validation cohort (*n* = 44)) and is complemented by a second one of primary tumours (PRI-Cohort; Survival cohort (*n* = 504)), and an additional cohort served for analysis of HRD status (*n* = 66).

The PRI-REC-Cohort consists of HGSOC patients who were treated between 2001 and 2015 at the Department of Gynaecology, Charité University Hospital Berlin. From these patients, paired tissue samples of their primary and recurrent lesions were available, which have been examined at the Institute of Pathology, Charité University Hospital Berlin. Patients were recruited in the OCTIPS project (Ovarian Cancer Therapy—Innovative Models Prolong Survival, www.octips.eu) and samples underwent central pathological review. Out of a total of 107 samples, cases were excluded if paired samples were not available (*n* = 22), samples were histologically inappropriate for our study (*n* = 13), or patients had already received neoadjuvant chemotherapy (NACT) (*n* = 10). After IHC evaluation, an additional 18 pairs had to be excluded: in five cases, TMA tissue of either the primary or the relapse samples was of insufficient quality and 13 pairs were excluded retrospectively because of falsely positive negative controls. Thus, 44 paired samples were available for IHC analyses (Supplementary Fig. 1a).

The PRI-Cohort consists of patients who likewise were treated and diagnosed at Charité and includes primary HGSOC specimens from patients followed between 2000 and 2021. Staining of p53 was used as an additional quality control for histotype [14]. In total, 504 patient samples could be included in our study (survival cohort), as they were considered eligible after reviewing for the above-mentioned inclusion and exclusion criteria (Supplementary Fig. 1b).

The HRD cohort consists of 66 HGSOC patients with mutated *TP53* (determined in 61 cases by immunohistochemical expression pattern and in 5 cases by molecular analysis). In 60 patients HRD status was determined using the HRD Plus Test Myriad Genetic Laboratories [15], in 5 patients the NOGGO GIS V1 assay [16] and one patient had *BRCA1* class 5 mutation according to the Oncomine BRCA Research Assay from ThermoFisher Scientific. For 60 patients IHC AHRR and for 62 patients IHC SFRP2 staining was informative.

Clinical data was obtained from the Tumour Bank Ovarian Cancer Network (www.toc-network.de) or the Charité Comprehensive Cancer Centre (https://cccc.charite.de). The conduct of this study was approved by the local ethics committee (EA1/051/18 and EA1/110/22). Clinicopathological parameters of the patient cohorts are summarised in Supplementary Table 1.

### Targeted RNA sequencing

Forty-eight paired formalin-fixed, paraffin-embedded (FFPE) tissue samples of primary and recurrent tumours of 24 patients from the PRI-REC-Cohort (*n* = 24/85) were prepared for HTG EdgeSeq analysis (provided by HTG Molecular Inc., Tucson, Arizona, USA).

For this purpose, H&E-stained large-area sections of the specimens were analysed by light microscopy, and sites with optimal tumour content were marked by an experienced gynecopathologist (S.D.E.). Corresponding unstained slides were forwarded to HTG, where tissue was scraped off and used for further processing. Workflow information is available on the HTG Web page (https://www.htgmolecular.com/systems/chemistry). Briefly, this method combines RNA extraction–free chemistry, a quantitative Nuclease Protection Assay and a qPCR for library preparation. The HTG EdgeSeq Oncology Biomarker Panel was used to measure the gene expression levels (mRNA) of 2,549 genes associated with tumour biology (Supplementary Table 2). Normalised libraries were analysed by Next Generation Sequencing (Illumina NextSeq Sequencer). After applying the previously stated inclusion and exclusion criteria, five of the sample pairs had to be excluded because of non-matching histotype (*n* = 3) or NACT (*n* = 2). Finally, 19 pairs (screening cohort, Supplementary Fig. 1a) could be included in further analysis (Supplementary Table 1).

### Immunohistochemistry

Immunohistochemical staining was performed semi-automatically on TMAs using a DISCOVERY XT/ULTRA autostainer (Ventana Medical Systems, Inc., Tucson, Arizona, USA). The following antibodies were used at dilutions previously tested on normal tissue: AHRR (1:3000, Abcam, Ref. No ab108518), COL5A2 (1:100, Sigma-Aldrich, Ref. No SAB4500385), FABP4 (1:1500, Abcam, Ref. No. ab92501), HMGCS2 (1:200, Abcam, Ref. No. ab137043), ITGA5 (1:600, Abcam, Ref. No. ab112183), SFRP2 (1:25, Abcam, ab92667), WNT9B (1:500, Abcam, Ref. No. ab151220). Positive and negative control tissues for antibody establishment were selected based on the manufacturer’s instructions and the Human Protein Atlas [17] (Supplementary Table 3). Universal negative controls of all TMAs were generated by omitting the primary antibody. If samples showed positivity (*H*-score ≥ 20, Supplementary Table 4), the affected samples were revised and excluded from all analyses. The stained TMAs were digitalised using the Pannoramic Slide Scanner (3D Histech, Budapest, Hungary).

### Methylation analysis

For 16 randomly selected HGSOC samples from the survival cohort, for which IHC data for AHRR and SFRP2 were available, a genome-wide methylation analysis was performed as previously described [18]. Briefly, tumour areas were punched out from the FFPE block for DNA extraction. Semi-automated DNA extraction was performed according to the manufacturer’s instructions (Maxwell RSC FFPE Plus DNA Purification Kit, Custom, Promega). DNA quantities were measured using Qubit HS DNA assay (Thermo Fisher Scientific). DNA restoration was performed using the Infinium HD FFPE DNA Restore Kit and methylation analysis was performed using the Illumina Infinium MethylationEPIC BeadChip. All methylation data pre-processing was conducted in R using various methods as implemented in the ChAMP package. Raw signals were loaded from the IDAT files using the minfi package [19, 20]. A number of CpG sites were filtered out: all SNP-related sites; multi-hit sites; and CpGs located on chromosomes X and Y. Lastly, the beta values of the remaining CpG sites were normalised using FunNorm [21] followed by BMIQ [22]. Next, we selected the normalised beta values for CpGs that passed the above filters and extracted from the annotated file provided by the Illumina the CpGs mapped to *AHRR* (138 CpGs) and *SFRP2* (41 CpGs) genes.

### Liquid chromatography-mass spectrometry (LC-MS)

LC-MS analysis of cell lines was performed with an EASYnLC-1200 system (Thermo Fisher Scientific) connected to a trapped ion mobility spectrometry quadruple time-of-flight mass spectrometer (timsTOF Pro2, Bruker Daltonik) with a nano-electrospray ion source (CaptiveSpray, Bruker Daltonik). Peptides were loaded on a 20-cm home-packed HPLC column (75-µm inner diameter packed with 1.9-µm ReproSil-Pur C18-AQ silica beads, Dr. Maisch). Peptides were separated over a 60 min gradient from 2 to 60% (0.5 min to 4%, 31.5 min to 20%, 15 min to 30%, 3 min to 60%, followed by a wash in 90% for 2 min and decrease to 50% in 7 min) in buffer B (0.1% formic acid and 90% ACN in LC-MS grade water) at 250 nl min^−1^. Buffer A consisted of 0.1% formic acid in LC-MS grade water. A column oven was used to keep the column temperature constant at 40 °C. For dia-PASEF analysis, we used a dia-PASEF method with 16 diaPASEF scans separated into 2 ion mobility windows per scan covering a 400–1200 *m*/*z* range by 25 Th windows and an ion mobility range from 0.60 to 1.43 Vs cm^−2^. The MS was operated in high sensitivity mode, with an accumulation and ramp time at 100 ms, capillary voltage set to 1400 V and the collision energy as a linear ramp from 20 eV at 1/K0 = 0.6 Vs cm^−2^ to 59 eV at 1/K0 = 1.6 Vs cm^−2^. MS raw file analysis was performed with DIA-NN [23] and described in detail in the supplementary methods.

### Statistical analysis

Statistical analysis was performed using IBM SPSS Statistics 26 (Armonk, NY, USA), R 3.5.2 (R Project for Statistical Computing, RRID:SCR_001905) and GraphPad Prism 9.0. Figures were created in SPSS, GraphPad Prism 9.0 and Biorender (https://app.biorender.com).

The count data was processed using the HTG parsing tool and scale-normalised using the trimmed mean of *M*-values (edgeR, RRID:SCR_012802). To perform a differential gene expression (DEG) analysis according to primary and recurrent disease, linear models with empirical Bayes moderation (LIMMA, RRID: SCR_010943) were fitted using a paired model design. Adjustment for multiple testing was carried out using the Benjamini-Hochberg method.

Exploratory analysis of the IHC protein level was done independently. The correlation of markers with clinical or pathological parameters was performed using the Chi² or Fisher’s exact test. For different levels between *H*-scores between tumour samples and for in vitro experiments we first assessed whether the data followed a normal distribution using the Shapiro–Wilk normality test and the *F*-test was employed for calculating variance between groups. Next, for paired samples, *p*‐values were determined with a paired *t*-test if data were normally distributed, and the non‐parametric Wilcoxon-signed rank test was used for values with a non‐normal distribution. For unpaired samples, *p*‐values were determined with an unpaired *t*-test if the data were normally distributed, while the Mann–Whitney test was applied for values with a non‐normal distribution. Correlation analysis between parameters was performed using Pearson’s r coefficient.

To determine the prognostic impact of the evaluated markers on patient survival, univariate Kaplan–Meier survival analysis (Log-rank test) and multivariate Cox regression analysis (age at diagnosis (≤60 versus >60), FIGO stage (FIGO I–II versus FIGO III–IV), residual tumour (R0 versus R1)) were performed. Overall survival (OS) was defined as the time from the day of pathologic diagnosis until the patient’s death, regardless of the cause. Progression-free survival (PFS) was defined as the time from diagnosis to occurrence of clinical progression, or recurrence, as measured by imaging. Optimal cutoffs for these calculations were obtained using the Cutoff Finder (https://molpathoheidelberg.shinyapps.io/CutoffFinder_v1/) and estimated based on OS [24]. *p*-values < 0.05 were considered statistically significant in two-sided testing. The statistical tests used for every figure were investigated to be appropriate and the data meet the assumptions of the tests.

### Other methods

Other methods are included in the Supplementary Information.

## Results

### Targeted RNA sequencing reveals differentially expressed genes in primary and recurrent HGSOC

**Fig. 1:**
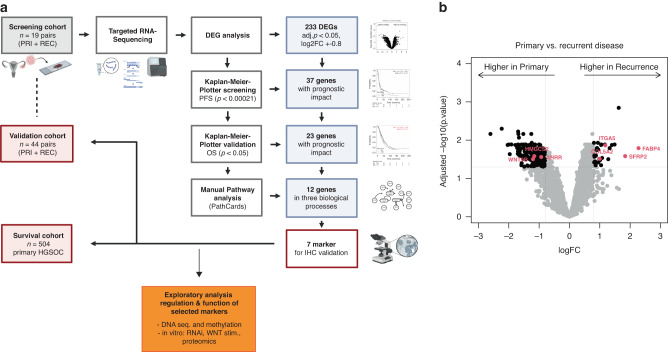
Biomarker selection procedure. **a** Scheme of study design. Created with BioRender.com, **b** Volcano plot of differential gene expression analysis according to primary and recurrent disease. For each gene, the log fold-change (log2FC) and the –log10 *p*-value are plotted. The dotted lines indicate the set cutoff values for further analysis (*p* < 0.05, |log2FC | > 0.8). Red dots mark the seven makers chosen for IHC.

A complex pipeline was designed to discover genes with potential key roles in HGSOC recurrence, validate the expression of their proteins, and to discover predictive biomarkers (Fig. 1a). To assess genes and biological pathways that might be associated with the process of tumour recurrence in HGSOC, we analysed 38 matched samples of primary and recurrent tumours from the PRI-REC-Cohort (*n* = 19, screening cohort). We found 373 genes to be differentially expressed between primary and recurrent samples after adjustment for multiple tests. As the cutoff criteria, genes with adjusted *p* < 0.05 and |log2FC | >0.8 were considered significantly differentially expressed and therefore applicable for further analysis (Fig. 1b). This approach resulted in 233 DEGs, of which 199 (85.4 %) were expressed higher in the primary tumours and 34 (14.6 %) showed higher expression in the recurrent samples (Supplementary Table 5).

### In silico analysis identifies a prognostic impact for a subset of genes and indicates a key role of extracellular matrix organisation

To further test whether the identified DEGs are of clinical significance in primary OC, we conducted a Kaplan–Meier Plotter survival analysis [25] for all 233 DEGs individually (Supplementary Table 6). In this analysis, 37 genes were identified as predictors for PFS of serous OC patients on the mRNA level (*p* < 0.0002; Bonferroni). Twenty-three of these 37 genes furthermore showed significant association with the OS (*p* < 0.05, Bonferroni, Supplementary Table 7). Twelve of these genes showed high expression in the primary tumour samples and 11 were upregulated in the recurrences (Supplementary Table 8).

To analyse which pathways might be affected by the DEGs in recurrent HGSOC, we performed gene ontology enrichment analysis. Analysis of the 11 genes upregulated in recurrent tumours with a significant prognostic impact, revealed enrichment of biological processes associated with collagen fibril organisation (GO:0030199), extracellular matrix organisation (GO:0030198) and supramolecular fibre organisation (GO:0097435). The same analysis of the 12 genes, that showed an upregulation in the primary tumours, did not lead to significant results.

To nail down genes most suitable for further analysis via IHC on tissue samples, we determined the biological function of all 23 DEGs via Pathcards.

This analysis revealed that 12 of the prognostic DEGs were clustering into three major biological functional groups which can be summarised as “Extracellular matrix organisation”, “Regulation of lipid metabolism by Peroxisome proliferator-activated receptor alpha (PPARalpha)” and “WNT signalling” (Supplementary Table 8). In conjunction of earlier studies [7] by our consortium and extensive literature review we focused on 7 genes. Three of these seven genes (*AHRR, HMGCS2*, and *WNT9B*) showed a higher expression in the primary tumours and have not been evaluated or described before in this context. Four genes (*FABP4, SFRP2, ITGA5*, and *COL5A2*) had an increased expression level in the recurrent tumours and were chosen as representatives for the above-mentioned pathways (Supplementary Figs. 2a–g and 3a–g).

### Compartment-dependent expression patterns in tumour samples of HGSOC

**Fig. 2:**
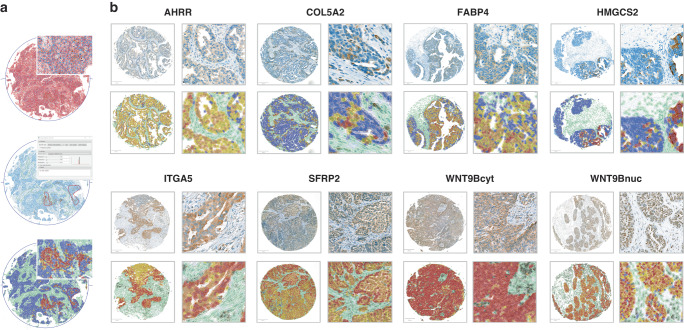
Immunohistochemical analysis. **a** Workflow of digital image analysis using QuPath**. b** Representative IHC-staining, cell detection and classification. Detected cells were classified and colour-coded using QuPath. Blue, negative tumour cells. Yellow, weakly stained tumour cells. Orange, moderately stained tumour cells. Red, strongly stained tumour cells. Green, cells of the tumour-microenvironment with increasing darkness according to intensity of protein expression. Images on the right are magnifications of areas in the pictures on the left.

To analyse the protein expression of these seven genes we used a specific pipeline employing digital pathological analysis of the IHC-stained TMA slides using QuPath (Fig. 2a). Antibody staining was detected in both primary and recurrent tumours and was mainly located in the cytoplasmic cell compartment with only WNT9B showing additional nuclear and ITGA5 showing sporadic membranous staining (Fig. 2b).

In addition to the detection in the tumoral compartment, the proteins were furthermore found in specific cells of the TME (Supplementary Fig. 4a–h). For example, FABP4 showed high expression in tumour surrounding adipocytes and COL5A2 was expressed in tumour-associated fibroblasts. While the expression of HMGCS2 was very clear in tumour cells, it was predominantly negative in the stroma (Supplementary Fig. 4d). Low *H*-scores determined by QuPath proved to be artificial after reviewing by a pathologist (E.T.T), hence the stromal expression of HMGCS2 was not included in the further analyses.

Protein expression distributions in the primary tumours (PRI-Cohort) showed a negative median *H*-score (0–1) for HMGCS2 (0.04) and low median *H*-scores (1–100) for FABP4, COL5A2, AHRR, cytoplasmatic WNT9B and ITGA5 (3.94; 7.2; 34.15; 80.81; 93.56) in the tumour compartment. Nuclear WNT9B expression (median *H*-score: 105.17) was moderate (*H*-score: 101–200) and median SFRP2 expression (*H*-score: 234.56) was strong (*H*-score: 201–300) (Supplementary Fig. 4a–h).

### AHRR and SFRP2 are differentially expressed in primary and recurrent tumour samples

**Fig. 3:**
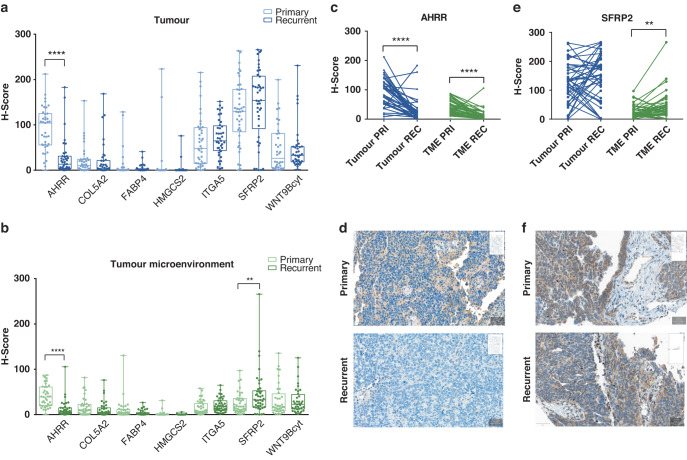
Differential expression on the protein level. **a** Boxplots of differential expression in the tumour compartment of primary (PRI) and recurrent (REC) samples, **b** Differential expression in the tumour microenvironment (TME). The 25–75th percentile of expression is represented by the boxes and the whiskers show minimum and maximum. Circles on the whiskers represent individual data points. Light colouring indicates the expression in primary samples, dark colouring for recurrent samples. **c** Before and After Plot of AHRR *H*-scores in the paired primary and recurrent HGSOC from the validation cohort (*n* = 39). **d** Example for AHRR protein expression in a primary and a recurrent sample, scale bars: 50 µm. **e** Distribution of SFRP2 *H*-scores in the paired primary and recurrent samples (*n* = 42). **f** Example for SFRP2 staining in a primary sample and a recurrence. Scale bars: 50 µm. Significance levels were determined using the Wilcoxon-signed rank test (**p < 0.01, ****p < 0.0001).

Next, we aimed to validate differences in gene expression between recurrent versus primary tumours on the protein level. Therefore, we analysed tumour compartments in which our gene set was differentially expressed, hence moving our study from a bulk level to a cellular level (Fig. 3a, b).

The median AHRR protein expression in the tumour cells in primary samples was 104.15 (*H*-score, range: 0.28–212,13, Supplementary Table 9) but 13.43 (H-score, range: 0–182.92) in the recurrences, showing a significantly higher AHRR expression in the tumour cells within the primary HGSOC group (*n* = 39 pairs, Wilcoxon *p* < 0.0001, Fig. 3c, d). Additionally, AHRR was higher expressed in the TME of primary samples when compared to the TME of recurrent samples (*n* = 39 pairs, Wilcoxon *p* < 0.0001, Fig. 3c.)

The contrary observation was made for SFRP2 (Fig. 3e, f), showing a higher median expression in the cells of recurrent tumour samples (32.59 *H*-score, range: 0.39–265.72) when compared to the matched primary ones (17.71 *H*-score, range: 0.23–97.06). This difference was statistically significant (*n* = 42 pairs, Wilcoxon *p* = 0.009) but restricted to the TME. In pairwise testing, median *H*-scores of the tumour compartment did not show expression differences for SFRP2 (*n* = 42 pairs, Wilcoxon *p* = 0.365). Interestingly, none of the other differentially expressed genes showed significant differences in the matched pairs at the protein level (Fig. 3a, b).

### Protein expression of FABP4, SFRP2 and cytoplasmatic WNT9B correlate with clinical and histological parameters

The associations between the expression levels of the seven IHC markers and clinicopathological parameters including age, FIGO stage, regional lymph node involvement (pN) and residual tumour (R) were analysed in the survival cohort. FABP4 protein expression in the tumour cells and the presence of regional lymph node metastases were inversely associated (*χ*²= 5.309, *p* = 0.021). N1 status was more common in patients with low FABP4 values.

Additionally, high expression of cytoplasmic (*p* = 0.049) and nuclear (*p* = 0.043) WNT9B in the TME was found in older patients (age at diagnosis >60 years) (Supplementary Table 10).

### Expression of AHRR and SFRP2 has a prognostic impact on HGSOC

The survival analysis was performed on primary tumours, using IHC data from the tumour compartment and the TME independently. Information on OS was available for all 504 patients and PFS data was available for 329 patients (65.3%). Median follow-up was 85.8 months (95% CI 70.1–101.5 months) in the survival cohort. As no cutoff values were primarily established for our markers, we defined them with the Cutoff Finder [24] as described in supplementary materials and methods (Supplementary Table 11).

**Fig. 4:**
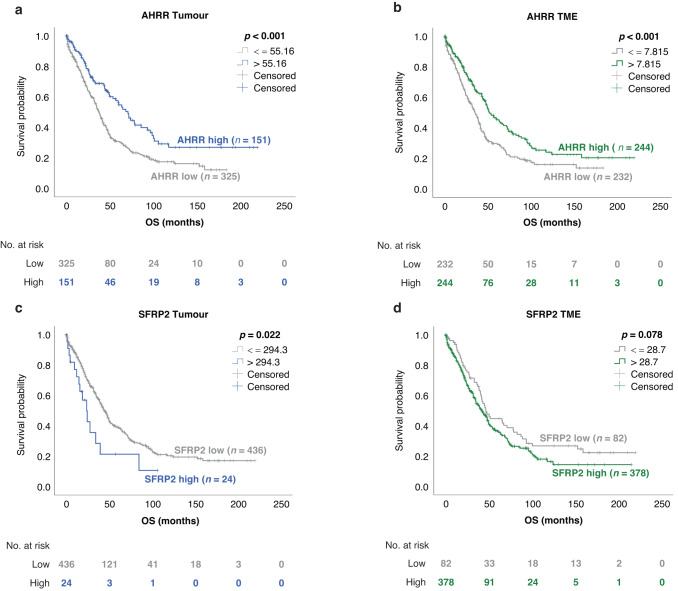
Kaplan–Meier survival curves according to the protein expression of AHRR and SFRP2 in the survival cohort. Cohorts were dichotomised by optimal *H*-score cutoffs. **a**, **b** OS according to AHRR expression in the tumour and the tumour-microenvironment (TME). **c**, **d** OS according to SFRP2 expression in the tumour and the TME. Significance levels were determined using the log-rank test.

For AHRR expression in the tumour cells, IHC data was available for 476 patients and the optimal prognostic cutoff out of 209/449 (46%) possible cutoffs was 55.16 (*H*-score). 325 patients (68.3%) showed a protein expression below this cutoff and 151 (31.7%) showed a protein expression above it. Median OS in the low expressing group was 37.4 months (95% CI 33.3–41.4 months) as opposed to 71.0 months (95% CI 58.2–83.9) for the high-expressing group (*p* < 0.001; Supplementary Table 11 and Fig. 4a).

When splitting the entire cohort according to the AHRR expression in the TME (cutoff: 7.815), a significantly superior OS within the high-expressing group was observed. In this group (*n* = 244, 51.3%) median OS was 52.0 months (95% CI 41.2–62.7 months) while in contrast a median OS time of 35.2 months (95% CI 29.8.–40.5 months) was seen in the group with low (*n* = 232, 48.7%) AHRR expression (*p* < 0.001, Fig. 4b). This positive prognostic impact also remained significant regarding the OS in the multivariate analysis for both tumour (HR 0.56, 95% CI 0.4–0.78, *p* = 0.001) and TME (HR 0.62, 95% CI 0.47–0.81, *p* < 0.001), verifying that AHRR is an independent prognostic marker in HGSOC, regardless of the compartment (Table 1).

**Table 1:** Multivariate Cox regression model for overall survival in the survival cohort with regard to AHRR and SFRP2 expression in the tumour cells and the TME.

AHRR	Tumour			TME		
	OS (*n* = 334)			OS (*n* = 334)		
Variable	HR	95% CI	*p*	HR	95% CI	*p*
Age > 60	1.23	0.943–1.603	0.128	1.29	0.989–1.683	0,.060
FIGO > II	2.68	1.187–6.067	**0.018**	2.59	1.146–5.870	**0.022**
Residual tumour	2.01	1.526–2.639	**<0.001**	2.01	1.527–2.639	**<0.001**
AHRR high	0.56	0.403–0.776	**0.001**	0.62	0.469–0.805	**<0.001**
**SFRP2**						
	**OS (*n*** = **316)**					
Variable	HR	95% CI	*p*			
Age > 60	1.23	0.937–1.620	0.136			
FIGO > II	2.40	1.059–5.420	**0.036**			
Residual tumour	2.11	1.593–2.799	**<0.001**			
SFRP2 high	2.57	2.566–1.499	**0.001**			

*TME* tumour microenvironment, *OS* overall survival, *n* number of patients, *HR* hazard ratio, *CI* confidence interval, *FIGO* Fédération Internationale de Gynécologie et d’Obstretique. Bold values indicate statistical significance *p* < 0.05.

Differences in PFS time according to tumoral AHRR expression were non-significant (*p* = 0.095, Supplementary Fig. 5a). The analysis of a correlation of stromal AHRR expression and PFS verified the OS results with a median PFS of 17.6 months (95% CI 15.2–20.1) for the AHRR low group and 22.6 months (95% CI 18.5–26.8) for the AHRR high group (*p* = 0.041, Supplementary Fig. 5b). Again, the prognostic value for PFS could be confirmed in the multivariate analysis (HR 0.72, 95% CI 0.55–0.96, *p* = 0.024, Supplementary Table 11).

A significant correlation with patient survival was also found for SFRP2, showing a lower median OS (23.8 months, 95% CI 14.7–32.9 months) for patients with a high expression of SFRP2 in their tumour cells (cutoff: 294.3; *n* = 24/460, 5.2%) as compared to the median OS time (44.0 months, 95% CI 39.1–48.9 months) for patients with a low expression (*n* = 436, 94.8%). This finding was statistically significant for the OS when SFRP2 expression in tumour cells was considered (*p* = 0.022, Fig. 4c), but not for expression in the TME (*p* = 0.078, Fig. 4d) or when PFS was considered for SFRP2 expression in tumour cells (*p* = 0.061, Supplementary Fig. 5c) or TME (*p* = 0.566, Supplementary Fig. S5d). Although the cutoff of 294.3 was only one out of 0.9% significant cutoffs found by the Cutoff Finder, its significance was retained in the multivariate analysis for the OS, indicating the independent negative prognostic impact of SFRP2 (HR 2.6, 95% CI 1.5–4.4, *p* = 0.001, Table 1). As SFRP2 acts as a regulator of the Wnt-signalling pathway and tumoral SFRP2 and stromal WNT9B showed an inverse impact on patient survival (Supplementary Table S11), we tested whether a combination of these two markers would be of further relevance. This analysis was performed for SFRP2 and cytoplasmatic WNT9B only, as data on WNT9Bnuc combinations were limited. Patients with SFRP2 + /WNT9Bcyt- status (*n* = 4) were found to have the worst OS when compared to the other three combinations, while patients with SFRP2-/WNT9Bcyt+ status (*n* = 128) had the longest survival interval (*p* = 0.035). The same observation could be made for the PFS, where survival was significantly better for patients with SFRP2-/WNT9Bcyt+ status (*p* = 0.021, Supplementary Fig. 6a, b).

In contrast to AHRR and SFRP2 other markers did not maintain significance in multivariate analysis (Supplementary Tab. 11) or could not be confirmed as being differentially expressed between primary and recurrent tumours on the protein level. COL5A2, HMGCS2 and ITGA5 expressed in the tumour compartment and cytoplasmatic as well as WNT9Bnuc in the stroma were favourable prognostic factors for OS (*p* < 0.05, Supplementary Fig. S7a–k) in univariate analysis. In multivariate analysis this significant correlation could be obtained only for stromal WNT9Bcyt (HR 0.64, 95% CI 0.47–0.88, *p* = 0.005) and WNT9Bnuc (HR 0.39, 95% CI 0.17–0.9, *p* = 0.028). For PFS cytoplasmatic WNT9B was found to be associated with better patient survival in the tumour cells (*p* = 0.029) as well as in the stroma (*p* = 0.017), which was confirmed (HR 0.67, 95% CI 0.48–0.92, *p* = 0.014) by multivariate analysis for the expression in the TME (Supplementary Fig. 8a–k and Supplementary Table 11). Interestingly, stromal FABP4 was revealed to correlate with a worse PFS (*p* = 0.043, Supplementary Fig. 8d, multivariate analysis: HR 1.41, 95% CI 1.06–1.86, *p* = 0.017).

Additionally, we wanted to check if the HRD status has any impact on the protein expression of AHRR and SFRP2. For this purpose, we used a third cohort for which HRD status was available. No association was detected between the HRD status and AHRR *H*-scores in tumour (*p* = 0.52, Supplementary Fig. 9a) or stroma cells (*p* = 0.7, Supplementary Fig. 9b) and SFRP2 *H*-score in tumour (*p* = 0.72, Supplementary Fig. 9c) or stroma cells (*p* = 0.87, Supplementary Fig. 9d).

### Exploratory analysis of the regulation and function of AHRR and SFRP2 in HGSOC

**Fig. 5:**
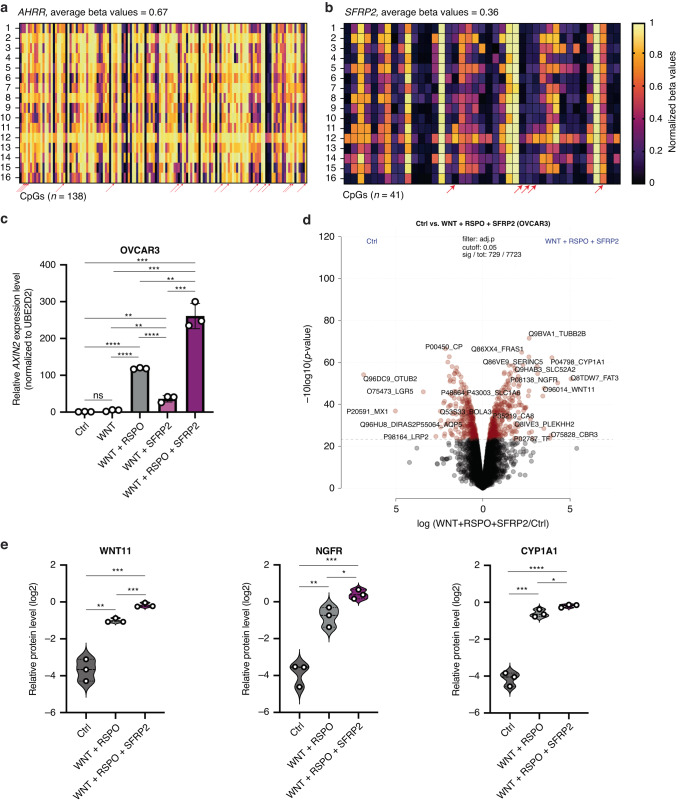
Exploratory analysis of the regulation and function of AHRR and SFRP2 in HGSOC. **a** Methylation levels (normalised beta values) in 138 CpGs associated with AHRR and **b** in 41 CpGs associated with SFRP2 in 16 patients with HGSOC for which also the protein expression of the two genes was available. With red arrows are marked the CpGs that negatively correlated with the *H*-score of the matching protein. **c** *AXIN2* mRNA level in OVCAR3 cells without treatment (Ctrl), and with WNT, WNT + RSPO, WNT + SFRP2 and WNT + RSPO + SFRP2. **d** Volcano plot of the pairwise proteomic comparison between the OVCAR3 control and OVCAR3 WNT + RSPO + SFRP2 stimulated cells. Significantly expressed proteins are highlighted in red (moderated two-sided *t*-test, FDR < 0.05). For both groups, the experiment was done in biological triplicates. **e** Relative protein level of WNT11, NGFR, and CYP1A1 in Control versus WNT + RSPO stimulated versus WNT + RSPO + SFRP2 stimulated OVCAR3 cell lines. Significance levels were determined using the unpaired *t*-test (ns = not significant, *p < 0.05, **p < 0.01, ***p < 0.001, ****p < 0.0001).

We wanted to understand the mechanism responsible for the dysregulated protein expression of AHRR and SFRP2 in HGSOC. First, we analysed DNA sequence alterations including mutations and copy number changes affecting these two genes in HGSOC samples from TCGA [9]. Out of 273 tumours analysed, 26 (9.5%) exhibited mutations or amplifications in *AHRR*, while 7 (3%) displayed copy number changes (amplifications and deletions) in *SFRP2* (Supplementary Fig. 10a). We also performed a comparative assessment of the prevalence of *AHRR* and *SFRP2* genomic sequence alterations between long-term and short-term survivors, but no significant differences were observed (Supplementary Fig. 10b). Second, we assessed the DNA-methylation profiles of both genes and performed genome-wide methylation profiling in 16 patients with HGSOC for which we had analysed AHRR and SFRP2 protein levels. We observed a synchronised hypermethylation of most of the 138 CpGs associated with *AHRR*, with an average methylation level of 0.67 (max. 1) (Fig. 5a and Supplementary Table 12). For *SFRP2*, which is overexpressed in HGSOC, we observed a synchronised hypomethylation of the 41 CpGs associated with this gene, with an average methylation level of 0.36 (Fig. 5b and Supplementary Table 13). Upon correlating the *H*-scores of AHRR and SFRP2 and methylation levels of the matching CpGs, we observed that 15 CpGs associated with *AHRR* (4 located in regulatory CpGs intragenic regions—body island) showed a significant negative correlation between their methylation and the protein levels (Supplementary Fig. 10c); and five CpGs associated with *SFRP2* (4 located in the transcription start site—TSS) showed a significant negative correlation between methylation and the protein levels (Supplementary Fig. 10d). Hence, it seems that the two genes are potentially dysregulated in HGSOC by aberrant focal DNA methylation.

To explore the function role of AHRR and SFRP2 protein expression in HGSOC, we tested their impact on cell proliferation using the DepMap Database. We observed that the knock-out of these genes compared to PAX8 (an established tumorigenic factor in HGSOC) [26] has no influence on tumour cell proliferation in 57 different epithelial ovarian cancer cell lines (Supplementary Fig. 11a). We analysed the basal expression of AHRR in five *TP53* mutated ovarian epithelial tumour cell lines and observed that AHRR has the highest expression in the EFO21 cell line (isolated from malignant ascites) (Supplementary Fig. 11b). Therefore, we selected this cell line for subsequent analysis to investigate the global proteome differences after AHRR knockdown (KD), using two different siRNAs (Supplementary Fig. 11c). Global proteome profiling by LC-MS analysis resulted in ~7,000 quantified proteins per single measurement and over 8000 proteins in total (Supplementary Fig. 11d and Supplementary Table 14). Albeit protein fold changes only revealed small differences, with one protein significantly upregulated (adj. *p*-value < 0.05) in the AHRR KD, the transcriptional regulator MLXIP (Supplementary Fig. 11e), pathway enrichment analysis based on the protein fold-change between AHRR KD versus control cells resulted in higher levels of proteins related to epithelial-to-mesenchymal transitions in AHRR KD cells, such as integrin cell surface, collagen degradation, collagen formation, degradation of the extracellular matrix and extracellular matrix organisation (Supplementary Fig 11f). This finding is in line with reports showing that low levels of AHRR are involved in invasion and migration in vitro and metastases in vivo in multiple cancers [27]. Collectively, the data support a potential tumour-suppressive role of AHRR, in strong agreement with our clinical samples data.

SFRP2 is a secreted modulator of WNT signalling [28] and was initially understood as a negative regulator of WNT signalling due to its ability to restrict the binding of Wnt ligands to their receptor [29]. More recent data show that SFRP2 can act as a negative or positive factor of WNT signalling, depending on the context [30]. Yet, its role in ovarian cancer is not defined. To functionally investigate the role of SFRP2 in ovarian cancer, we cultivated five epithelial ovarian cancer cell lines in the absence and presence of extracellular WNT effectors, including WNT3 (WNT) alone; WNT and RSPO1 (RSPO); WNT and SFRP2 (recombinant human protein); and WNT, RSPO and SFRP2 and tested the level of *AXIN2* as a WNT/β-catenin target gene [31]. We found that all cell lines were WNT-responsive, as the addition of WNT and the co-ligand RSPO to the medium-activated *AXIN2*. In all five cell lines, SFRP2 had no significant inhibitory effect on WNT signalling, and in OVCAR3 cells (isolated from malignant ascites) SFRP2 significantly further activated *AXIN2* compared to the activation provided by WNT plus RSPO (Fig. 5c and Supplementary Fig. 11g), suggesting a role of SFRP2 as a WNT activator in a subset of ovarian cancers. Next, we performed MS-based proteomic profiling to study the global effects of WNT activation and the consequence of SFRP2 addition. Our analysis resulted in ~7500 quantified proteins per single replicate (Supplementary Fig. 11h and Supplementary Table 15). By comparing OVCAR3 control to OVCAR3 with WNT + RSPO + SFRP2, we observed pronounced global protein level changes with indications of WNT signalling activation, with significant overexpression of WNT11 (Fig. 5d), a non-canonical WNT signalling molecule with a role in cancer [32]. Among the most upregulated proteins with SFRP2 addition, besides WNT11, were nerve growth factor and its receptor (NGFR) and CYP1A1 which showed a more significant upregulation in WNT + RSPO + SFRP2 versus control or WNT + RSPO (Fig. 5e). Previous studies have shown that NGFR is linked to WNT/β-catenin signalling and activates ovarian cancer tumour spread [33], while CYP1A1 was shown to be overexpressed in ovarian cancer clinical samples and to play a carcinogenic role [34]. Taken together, our functional investigations further support a tumour-suppressive role of AHRR and an oncogenic function of SFRP2 in ovarian epithelial cancer.

## Discussion

In the present study, we interrogated information on DEGs in primary and recurrent HGSOC in the quest for clinically applicable biomarkers. Targeted RNA sequencing and in silico survival analysis revealed a prognostic impact of 23 genes. Further IHC analysis of seven markers (AHRR, COL5A2, FABP4, HMGCS2, ITGA5, SFRP2 and WNT9B) demonstrated significant differences for AHRR being lower and SFRP2 being higher expressed in recurrent HGSOC. To test if these markers could serve as prognostic biomarkers already in primary tumours survival analysis was performed in a large cohort of 504 patients with primary HGSOC and demonstrated a favourable prognostic significance of high AHRR and low SFRP2 expression. In vitro data further supported the tumour suppressor role of AHRR and the pro-tumorigenic role of SFRP2.

Previous studies have shown that the modulation of the extracellular matrix and the tumour immune microenvironment are important mechanisms by which ovarian cancer cells might regulate tumour progression in recurrent or metastatic OC [6, 11, 12, 35]. Kreuzinger et al. defined primary and recurrent HGSOC samples as immune active or immune silent based on an unsupervised clustering method following RNA sequencing. Immune active recurrent samples showed an upregulation of genes involved in the remodelling of the extracellular matrix (e.g., *POST, COMP, COL5A2*) or genes like *SFRP2* and *ADH1B*. Primary samples with an immune active status additionally overexpressed genes associated with adipose tissue remodelling (e.g., *FABP4, GPD1, PLIN1*) [7]. Further studies on the immune modulation [6, 11, 12, 35] and angiogenesis [13] in primary and recurrent OC were based on the evaluation of specific IHC markers such as MVD, VEGF-A, MHC1, MHC2, PD-L1, IDO or different lymphocyte markers and showed increased expression of CD4+, MHC1 [12], PD-L1 [6, 35] and IDO [6], as well as higher levels of regulatory T cells [11] in recurrent samples.

In our study, we took a novel approach. It comprises an analysis of DEGs in primary and recurrent HGSOC and complements the aforementioned studies by adding a biomarker selection procedure based on in silico survival analysis and evaluation on a large cohort of primary tumours. During this process, we identified AHRR and SFRP2 as being consistently differentially expressed and with a significant prognostic potential for predicting OS.

The repressor of the aryl-hydrocarbon receptor (AHRR) showed an expression profile in line with the current understanding of it as a tumour suppressor [36] being higher expressed in primary tumours versus relapse. Patients with high AHRR expression displayed a significant increase in OS.

AHRR negatively regulates AHR signalling by competing with the AHR complex for its interaction with the aryl-hydrocarbon receptor nuclear translocator, thereby suppressing AHR signalling [37]. AHR is consistently active in a variety of human cancers and has several oncogenic functions [38], among them the mediation of pro-tumorigenic immunosuppression [39] and epithelial-mesenchymal transition [40]. AHR also dysregulates BRCA1 [41] expression, a crucial tumour suppressor in HGSOC and is additionally correlated with an unfavourable prognosis in OC [42]. Nevertheless, we could not detect an association of protein expression of AHRR with HRD status. Interestingly, AHR signalling was recently found to be the highest-ranking dysfunctional pathway in FIGO stage IV serous OC [43]. Therefore, regulation of AHR activation via AHRR expression might indicate an important anti-oncogenic mechanism in primary tumours [44]. In favour of this hypothesis there are recent observations in breast cancer showing that high *AHRR* mRNA levels are associated with favourable metastasis-free survival [45]. On the contrary, loss or downregulation of AHRR is proposed to have pro-oncogenic functions and drives tumour progression. To strengthen this hypothesis, we performed additional exploratory studies regarding the mechanism of AHRR downregulation and downstream function in OC. Genome-wide methylation analysis showed *AHRR* hypermethylation in HGSOC, and 15 CpGs showed an inverse correlation with matched protein expression. These 15 CpGs are located in the gene body, and four of them in intragenic CpG islands. Hypermethylation of gene-body CpGs can induce activation of transcription, yet the role of intragenic CpG islands is more complex and not fully understood. These regions, if hypermethylated can also inhibit gene transcription and have functions similar to promotor or enhancer regions [46]. Downstream analysis of AHRR function showed no impact on cell proliferation, rather proteomic studies revealed upregulation of proteins associated with epithelial-mesenchymal transition following AHRR KD. This observation is in line with previously published data showing that low AHRR levels in different cancers could induce resistance to apoptosis and increases the migratory potential of tumour cells [27, 36].

*SFRP2*, the secreted frizzled-related protein 2, encodes an extracellular Wnt-signalling modulator, which directly interacts with Wnt proteins. SFRP2 can act as a Wnt-suppressing or -activating factor in different tissue contexts and, therefore, exert tumour-suppressive or -promoting roles. Wnt-activating and tumour-promoting roles of SFRP2 have previously been identified in colon and lung cancer, as well as in glioblastoma [47–49], and our study suggests a similar role in ovarian cancer. In five ovarian cancer cell lines, SFRP2 did not inhibit WNT signalling and more interestingly, in one cell line, SFRP2 activated WNT signalling. We confirmed this activation by performing MS spectrometry, observing the upregulation of WNT11, a member of the non-canonical WNT signalling pathway. Furthermore, we observed an upregulation upon SFRP2 stimulation of other proteins with oncogenic function in HGSOC such as NGFR and CYP1A1, suggesting a potentially more complex tumorigenic function of SFRP2 in HGSOC.

Our IHC analysis also points to a pro-tumorigenic function of SFRP2. SFRP2 expression was higher in the stromal compartment of the recurrences and furthermore correlated with worse survival. These findings are in line with a study from Mariani et al. [50] where *SFRP2* was found to be overexpressed in OC bowel metastases when compared to primary samples. In comparison to our study, they could only show the differential expression on the RNA level, but also described the association of high *SFRP2* RNA expression with poor overall survival in a HGSOC patient cohort [50]. *SFRP2* gene expression was furthermore significantly upregulated in HGSOC specimens following NACT [51, 52] and Yuan et al. report *SFRP2* as part of a cluster consisting of nine key genes, able to predict high-risk patients with worse survival probability [53]. These results suggest a potential role of SFRP2 as a biomarker for poor survival.

Mechanisms of SFRP2 overexpression are not yet clear as the findings of several previous studies indicate a downregulation of *SFRP2* in OC due to methylation of its promotor region [54–56] and *SFRP2’s* potential to inhibit migration of OC cells [57]. In order to clarify the mechanism of *SFRP2* regulation in HGSOC, we performed genome-wide DNA-methylation analysis of patient samples. Our data revealed that *SFRP2* is hypomethylated in HGSOC and 4 CpGs located in the TSS showed a negative correlation to the matching protein *H*-score.

For SFRP2 our protein-based analysis is consistent with the results obtained by using the Kaplan–Meier-Plotter on mRNA levels. Surprisingly, high AHRR expression indicated increased OS in our own study, but the opposite direction was predicted by in silico survival analysis. The mRNA-based Kaplan–Meier analysis is furthermore puzzling since *AHRR* expression consistently indicated a tumour suppressor function in our own RNA and protein analysis in the PRI-REC cohort. Regarding prognosis, congruent results were obtained from our survival analysis of patients with primary HGSOC. These differences might be explainable by the fact that *TP53* mutation status was not available for all patients diagnosed with serous tumours in the datasets included in the Kaplan–Meier-Plotter [25], which is why the in silico analysis also included a small number of low-grade serous tumours. Additional differences might be caused by the circumstance that microarray gene expression data is obtained from the bulk analysis, which leads to the admixture of signals from the tumour and the stromal compartments [58].

The small sample size of our PRI/REC cohort and of our validation cohort could lead to data overfitting. We would like to point out that paired primary and relapse samples are difficult to obtain and we conducted our analysis with the best available sample size. Furthermore, we reduced the risk of overfitting the data by using conventional statistical methods as opposed to machine learning approaches and by confirming our findings in independent cohorts and by different methods. We used a highly standardised method for digital image analysis, which allows the separate analysis of the tumour and the TME, possibly leading to more precise results. Nevertheless, IHC analysis has some biases too, e.g., tissue processing or antibody specificity. To clarify the question on the correlation of mRNA and protein in our survival cohort, RNA sequencing data would have been needed ideally combined with tissue from relapse which is a limitation of our study. As we used optimised cutoffs for survival analysis, additive prospective clinical research needs to validate the prognostic implication of the markers on the survival of HGSOC patients.

As a conclusion, we present a highly explorative analysis of the differences in RNA and protein expression between primary and recurrent HGSOC, which lays the foundation for further research. Our results show that *AHRR* (tumour suppressor) and *SFRP2* (oncogene) are differentially expressed between primary and recurrent tumours both on the gene and the protein level and furthermore act as protein-based prognostic markers for HGSOC. Overall, the present study provides novel insights into the potential biological pathways and protein signatures involved in the process of recurrence and poor survival in HGSOC, *en passant* coming up with two potential prognostic biomarkers for advanced HGSOC.

### Supplementary information

supplementary tables

supplementary figures and methods

## Data Availability

The datasets used during this study are available from the corresponding author upon reasonable request.
