# Supplementary material for: AHRR and SFRP2 in primary versus recurrent high-grade serous ovarian carcinoma and their prognostic implication
Source: Br J Cancer. 2024 Feb 9;130(8):1249–60. doi: 10.1038/s41416-023-02550-1 (PMC11014847; doi:10.1038/s41416-023-02550-1)
Supplement: Supplementary file 2 — supplementary figures and methods [file 41416_2023_2550_MOESM2_ESM.docx]

**Supplementary Information**

**AHRR and SFRP2 in primary versus recurrent high-grade serous ovarian carcinoma and their prognostic implication**

**Nanna Monjé**, **Mihnea P. Dragomir**, Bruno V Sinn, Inga Hoffmann,

Anuar Makhmut, Tincy Simon, Catarina A Kunze, Jana Ihlow,

Wolfgang D Schmitt, Jonathan Pohl, Iris Piwonski, Sofya Marchenko, Carlotta Keunecke,

Teodor G. Calina, Francesca Tiso, Hagen Kulbe, Caroline Kreuzinger,

Dan Cacsire Castillo-Tong, Jalid Sehouli, Elena I Braicu, Carsten Denkert,

Silvia Darb-Esfahani, Kirsten Kübler, David Capper, Fabian Coscia,

Markus Morkel, David Horst, Christine Sers, Eliane T Taube

**Supplementary Figures** Page 2

**Supplementary Tables** Page 18

**Supplementary Methods** Page 19

**Supplementary References** Page 24

**Supplementary Figures**

**Supplementary Figure S1**

**
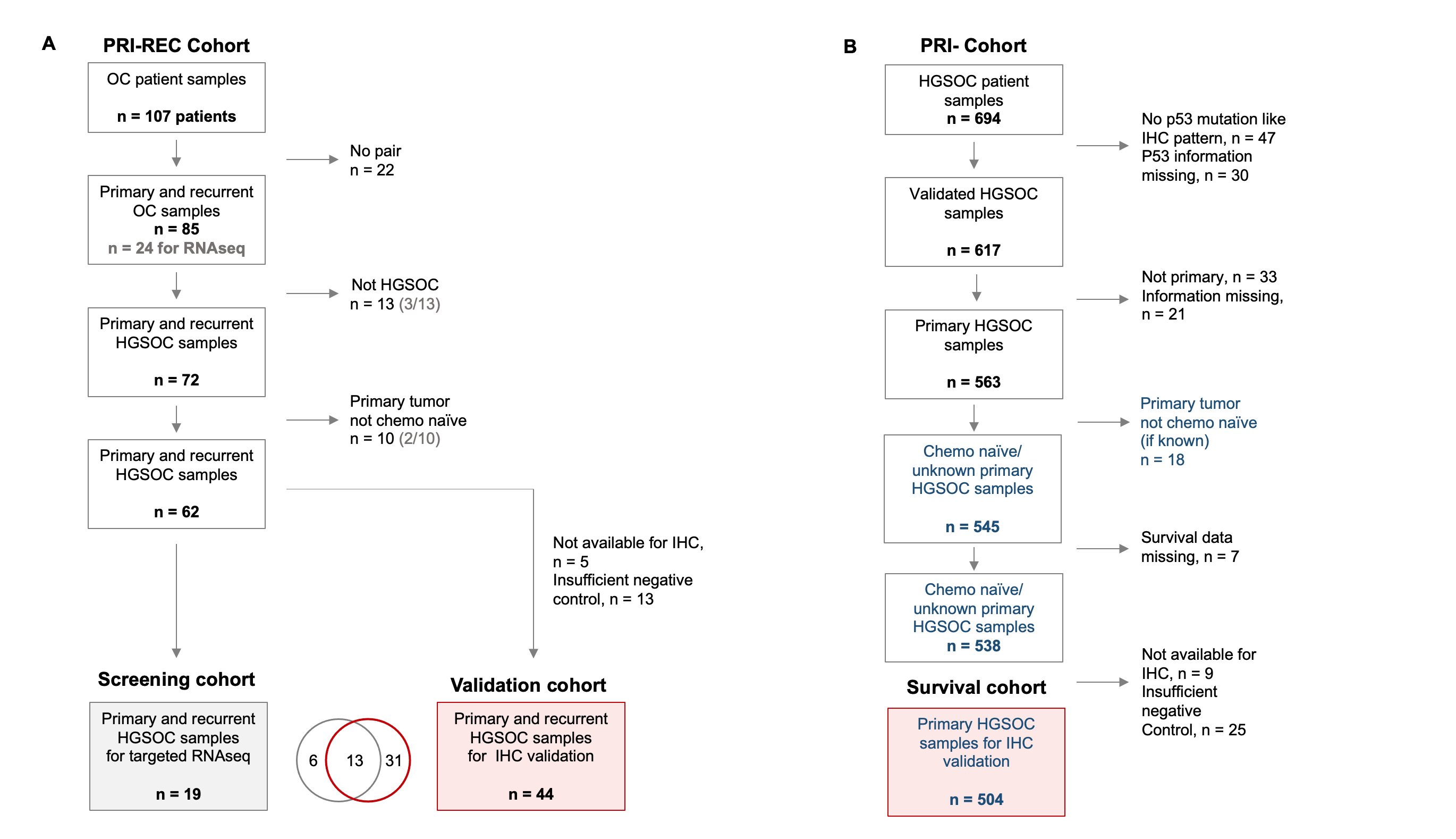
**

**Figure S1:** **Flowchart of the patient cohort compilation.** **A.** Cohort of matched primary and recurrent samples, **B.** primary HGSOC cohort, Number of included patients is stated in the boxes, reasons for exclusion are given on the right.

**Supplementary Figure S2**


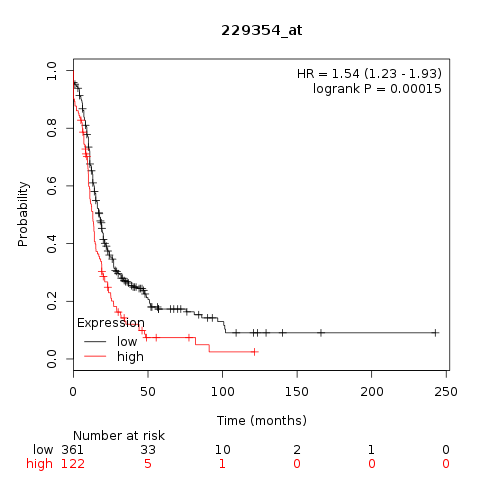


AHRR


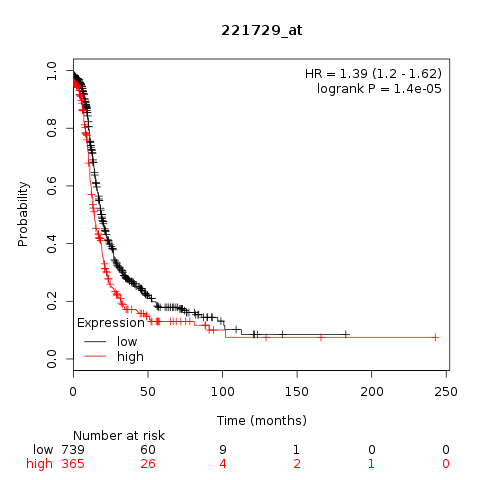


COL5A2


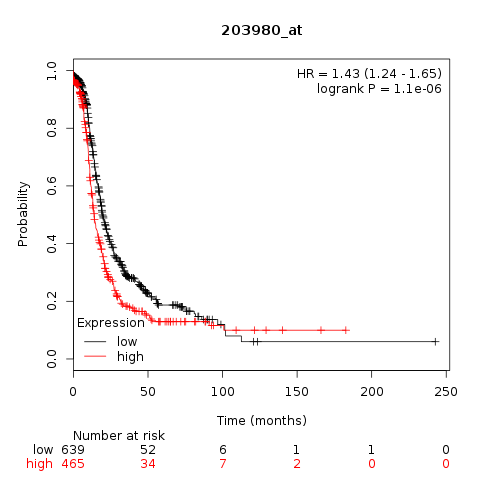


FABP4


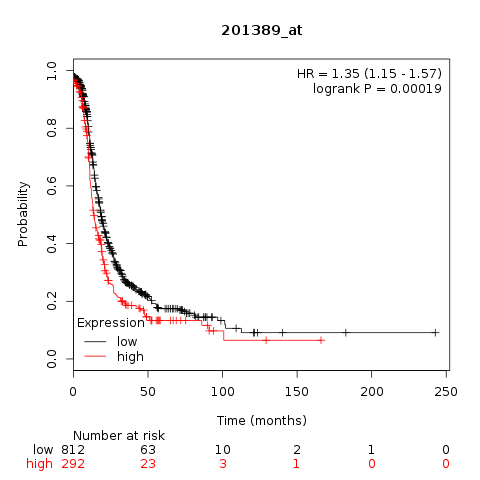


ITGA5


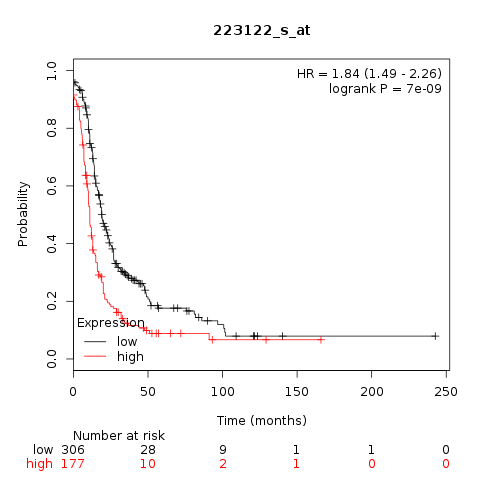


SFRP2


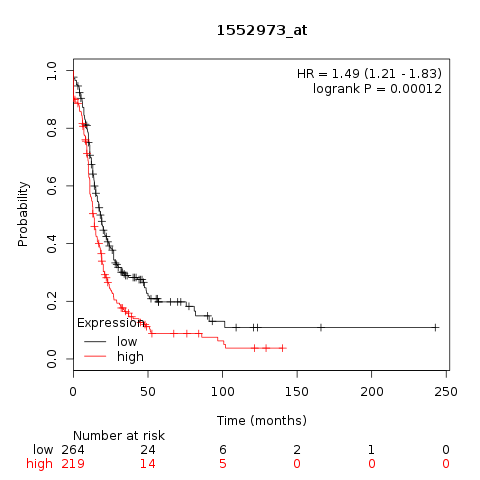


WNT9B


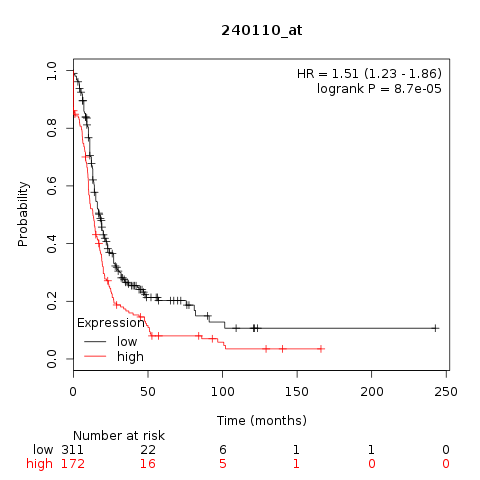


HMGCS2

**A**

**B**

**C**

**E**

**F**

**G**

**D**

**Progression-free survival**

**Figure S2: Survival plots generated with the Kaplan-Meier Plotter (https://kmplot.com/analysis/)** Analysis of the prognostic impact on progression-free survival (PFS) of the expression (mRNA) of *AHRR* (**A**), *COL5A2* (**B**), *FABP4* (**C**), *HMGCS2* (**D**), *ITGA5* (**E**), *SFRP2* (**F**), *WNT9B* (**G**). Classification in high and low expressing groups with auto select best cutoffs.

**Supplementary Figure S3**

AHRR

COL5A2

FABP4

ITGA5

SFRP2

WNT9B

HMGCS2

**A**

**B**

**C**

**E**

**F**

**G**

**D**

**Overall survival**


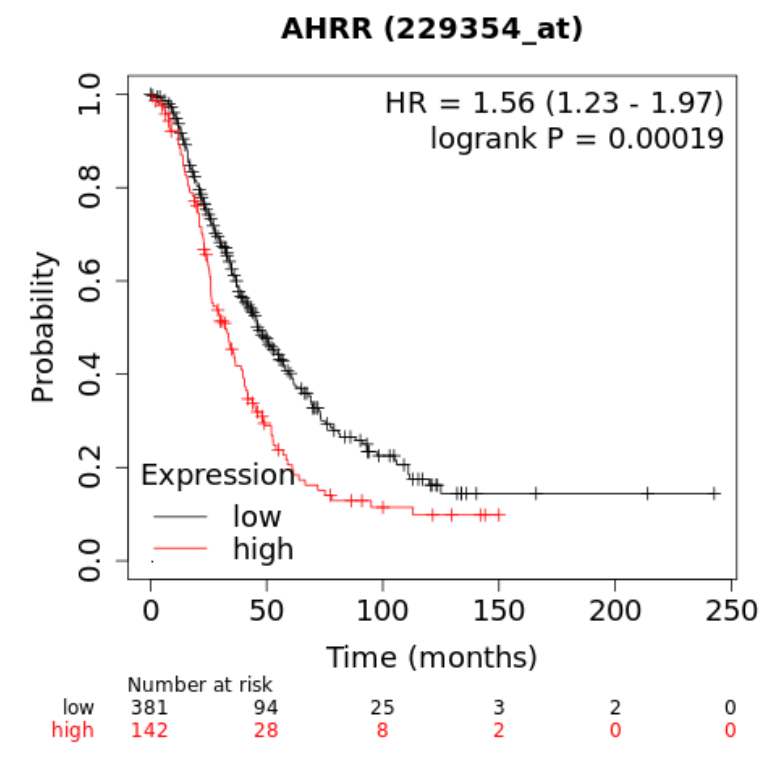

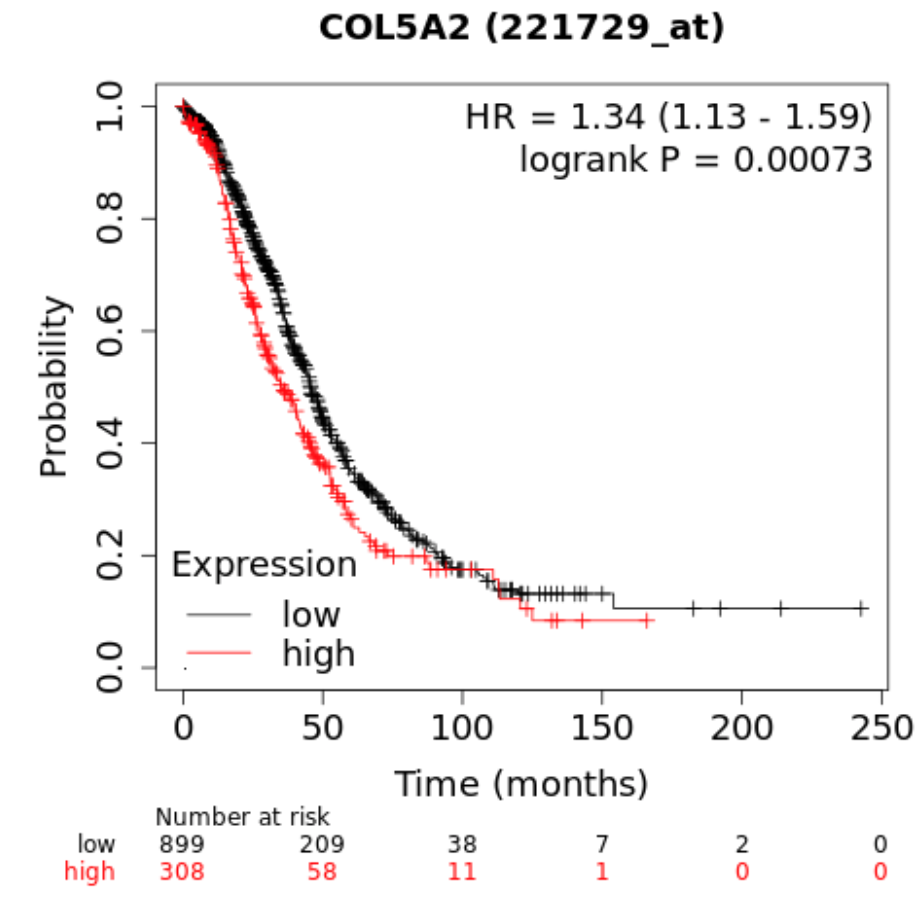

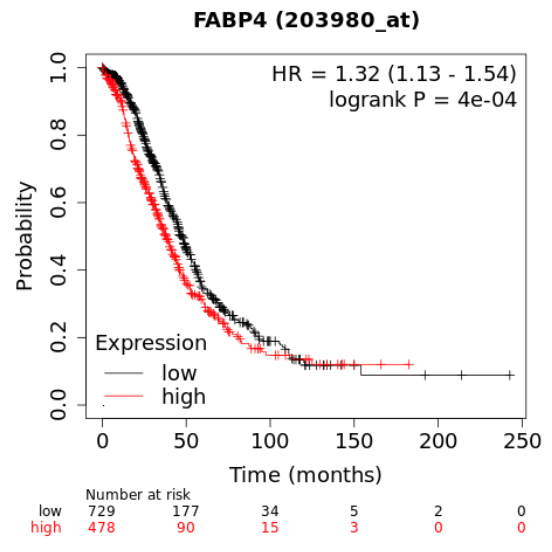

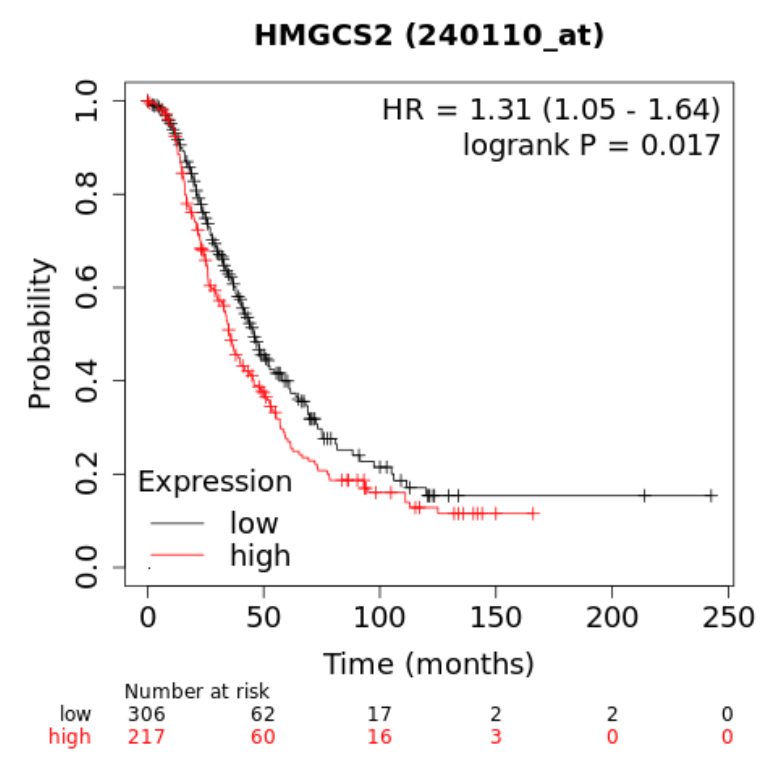

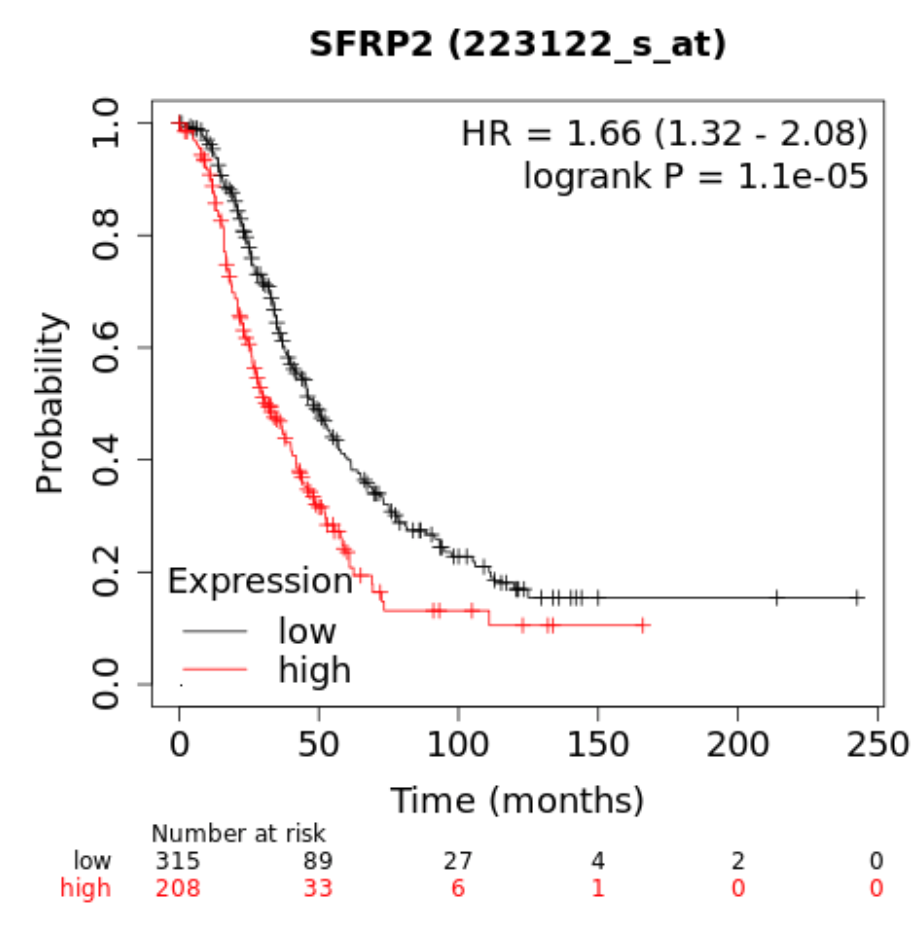

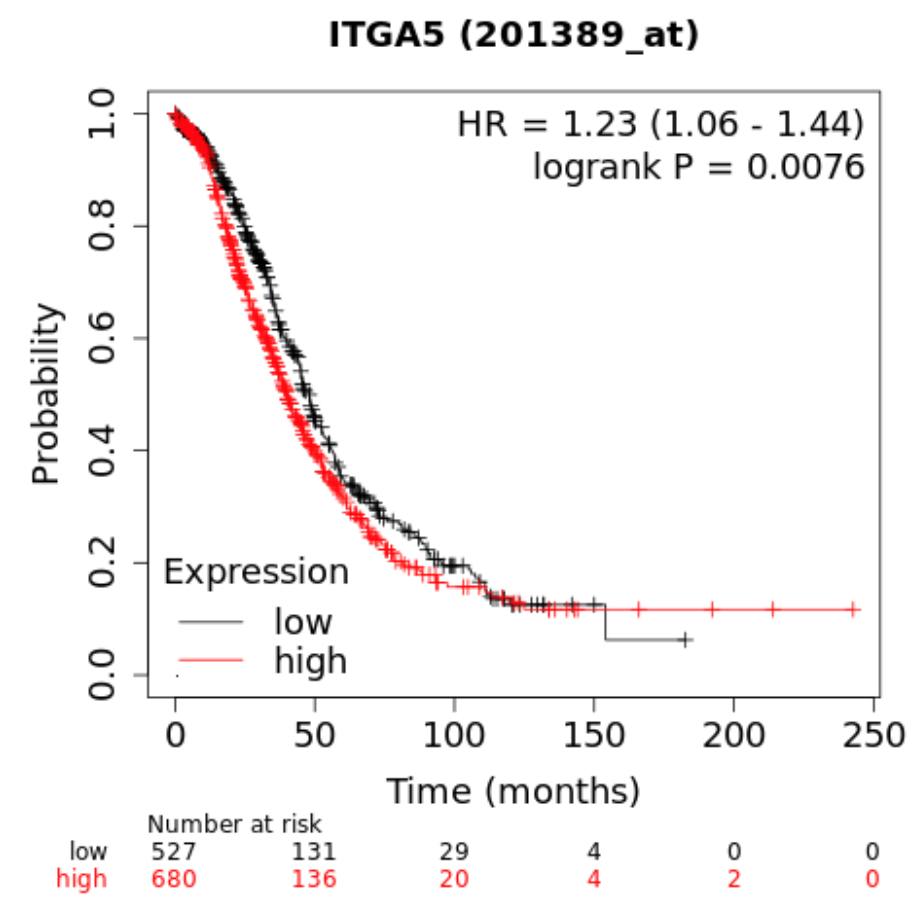

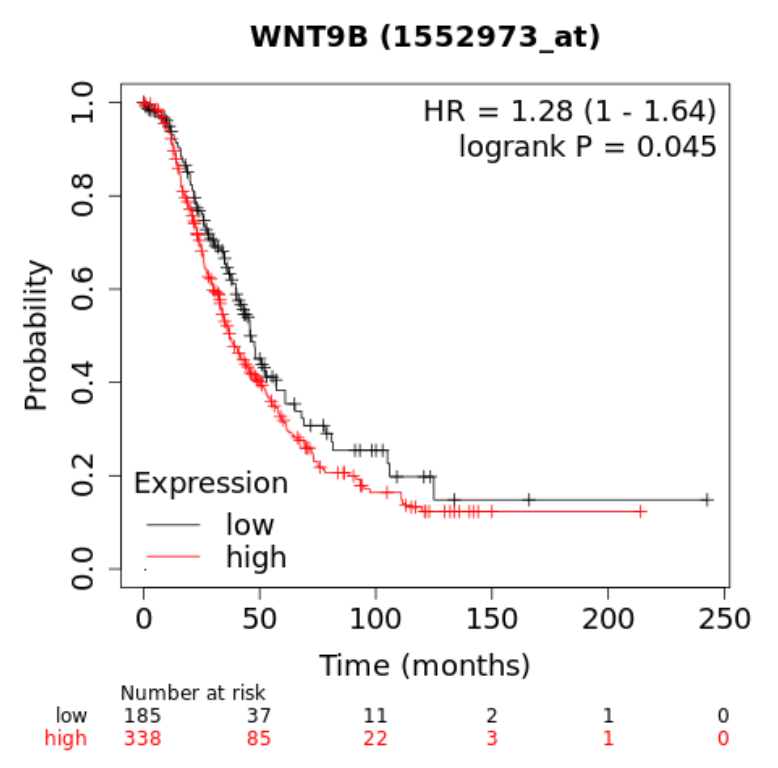


**B**

**C**

**Figure S3: Survival plots generated with the Kaplan-Meier Plotter (https://kmplot.com/analysis/)** Analysis of the prognostic impact on overall survival (OS) of the expression (mRNA) of *AHRR* (**A**), *COL5A2* (**B**), *FABP4* (**C**), *HMGCS2* (**D**), *ITGA5* (**E**), *SFRP2* (**F**), *WNT9B* (**G**). Classification in high and low expressing groups with auto select best cutoffs.

**Supplementary Figure S4**

**A**

**B**

**C**

**D**

**E**

**F**

**G**

**H**

**Figure S4: Protein expression in primary HGSOC**. Distribution of H-Scores in the tumor compartment (blue) and the tumor microenvironment (TME) (green) in primary HGSOCs from the survival-cohort. Solid lines illustrate the median, dashed line the selected best cutoff. **A**: AHRR; median H-Score Tumor: 34.15, median H-Score TME: 8.31, **B**: COL5A2; median H-Score Tumor: 7.2; median H-Score TME: 9.14 **C**: FABP4; median H-Score Tumor: 3.94; median H-Score TME: 3.11, **D**: HMGCS2; median H-Score Tumor: 0.04; median H-Score TME: 0.05, **E**: ITGA5; median H-Score Tumor: 93.56; median H-Score TME: 23.25, **F**: SFRP2; median H-Score Tumor: 234.56; median H-Score TME: 78.61, **G**: WNT9Bcyt; median H-Score Tumor: 80.81; median H-Score TME: 32.97, **H**: WNT9Bnuc; median H-Score Tumor: 105.17; median H-Score TME: 96.03.

**Supplementary Figure S5**

**
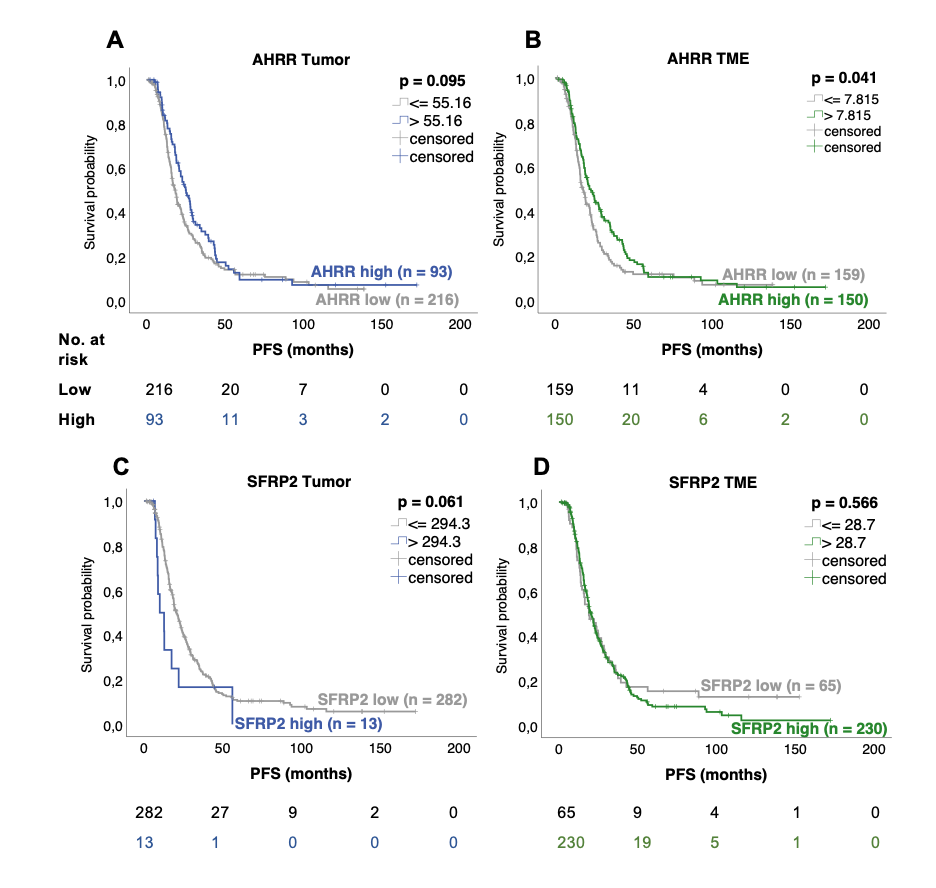
**

**Figure S5: Kaplan-Meier survival curves (PFS**). Analysis of the prognostic impact of protein expression on progression-free survival in the tumor compartment (blue) and the tumor microenvironment (TME) (green) in primary HGSOCs from the survival-cohort. Cohorts were dichotomized by optimal H-Score cut-offs. **A, B**: PFS according to AHRR. **C, D**: PFS according to SFRP2. P-value: Log-rank test.

**Supplementary Figure S6**


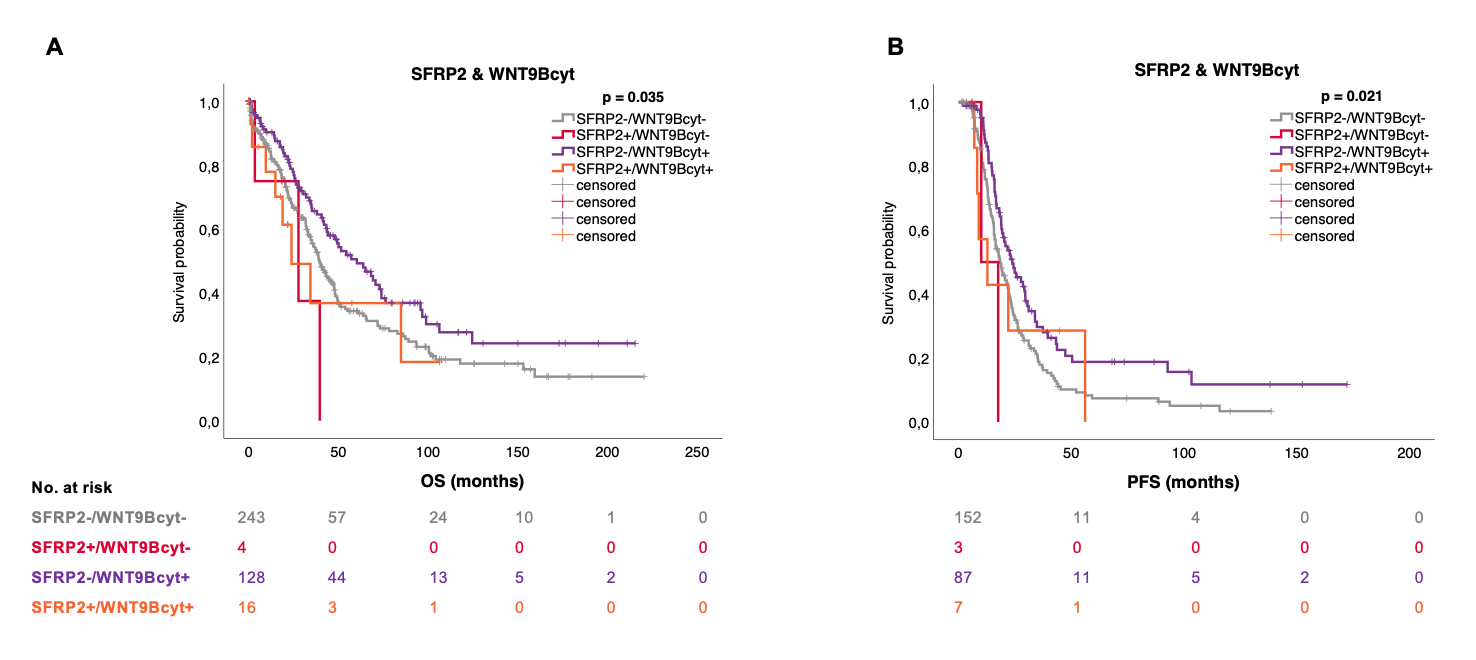


**Figure S6: Kaplan-Meier survival curves according to the combination of tumoral SFRP2 and WNT9Bcyt in the stroma.** Cohorts were dichotomized by optimal H-Score cut-offs. **A**: Overall survival (OS), **B:** Progression free- survival (PFS). P-value: Log-rank test.

**Supplementary Figure S7**


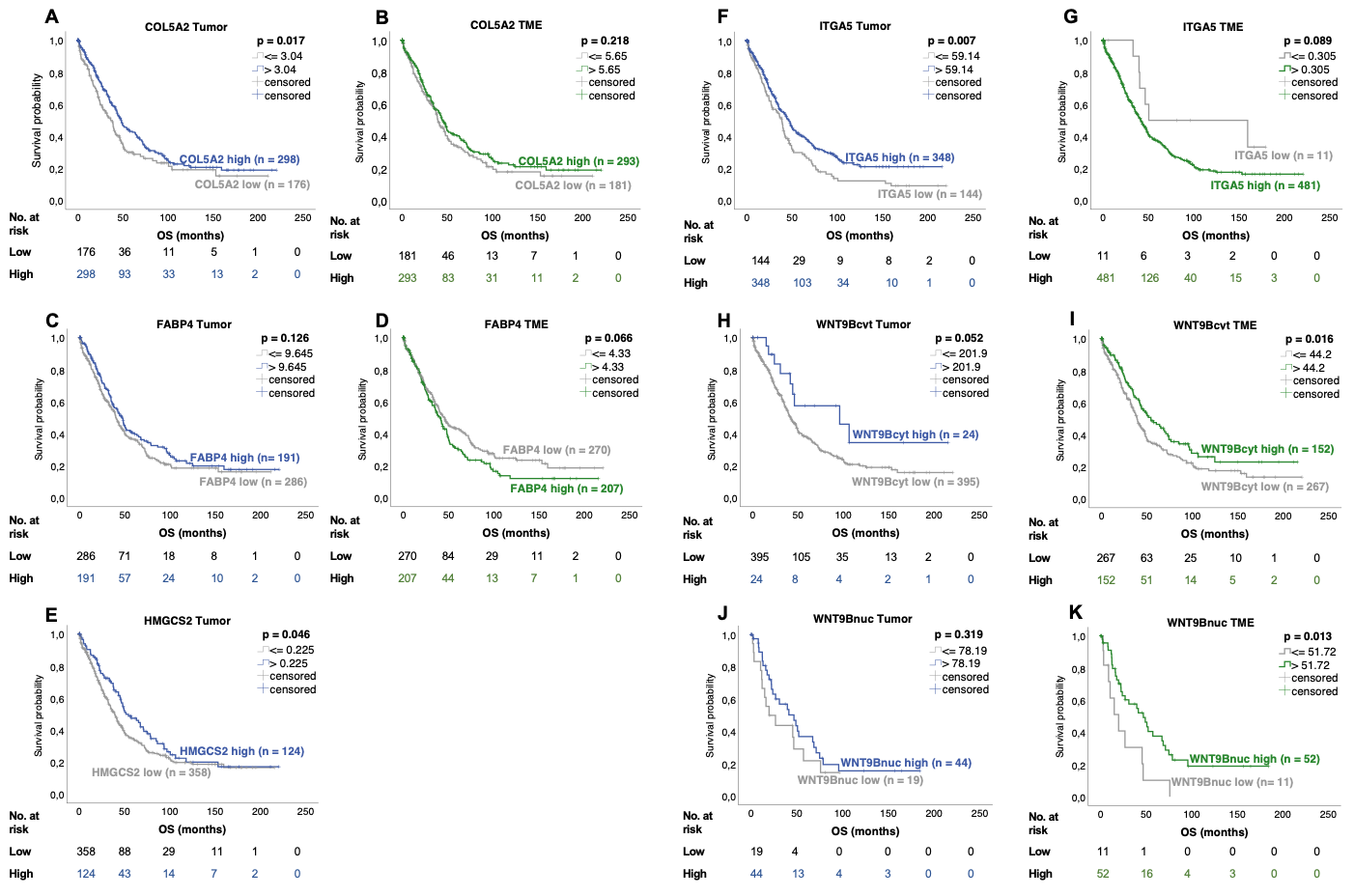


**Figure S7: Kaplan-Meier survival curves (OS).** Analysis of the prognostic impact of protein expression on overall survival in the tumor compartment (blue) and the tumor microenvironment (TME) (green) in primary HGSOCs from the survival-cohort. Cohorts were dichotomized by optimal H-Score cut-offs. **A, B**: OS according to COL5A2. **C, D**: OS according to FABP4. **E**: OS according to HMGCS2. **F, G**: OS according to ITGA5. **H, I**: OS according to cytoplasmic WNT9B. **J, K**: OS according to nuclear WNT9B. (P-value: log rank test, overall survival (OS)).

**Supplementary Figure S8**


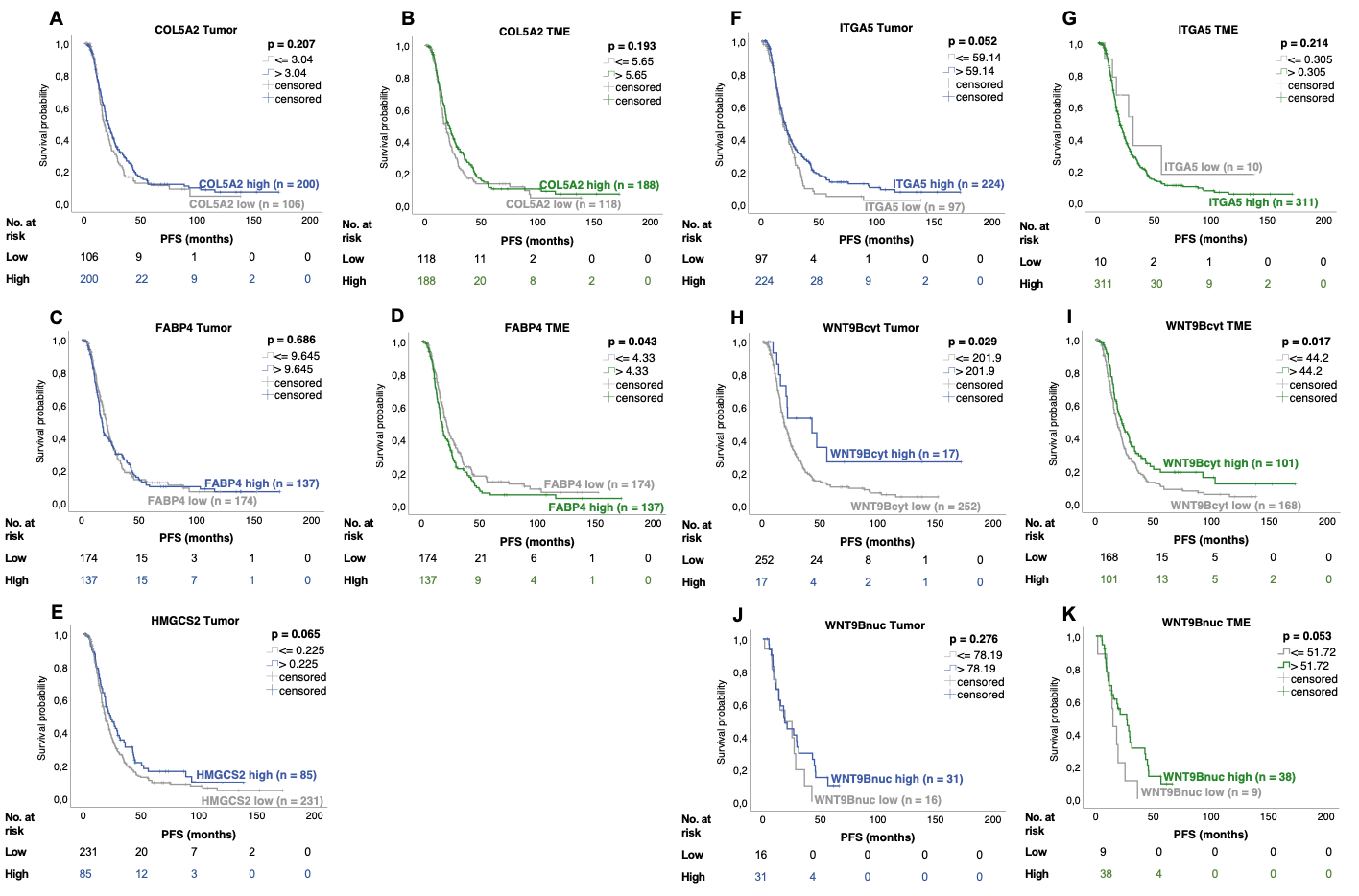


**Figure S8: Kaplan-Meier survival curves (PFS**). Analysis of the prognostic impact of protein expression on progression-free survival in the tumor compartment (blue) and the tumor microenvironment (TME) (green) in primary HGSOCs from the survival-cohort. Cohorts were dichotomized by optimal H-Score cut-offs. **A, B**: PFS according to COL5A2. **C, D**: PFS according to FABP4. **E**: PFS according to HMGCS2. **F, G**: PFS according to ITGA5. **H, I**: PFS according to cytoplasmatic WNT9B. **J, K**: PFS according to nuclear WNT9B. (P-value: log rank test, progression free survival (PFS)).

**Supplementary Figure S9**

**
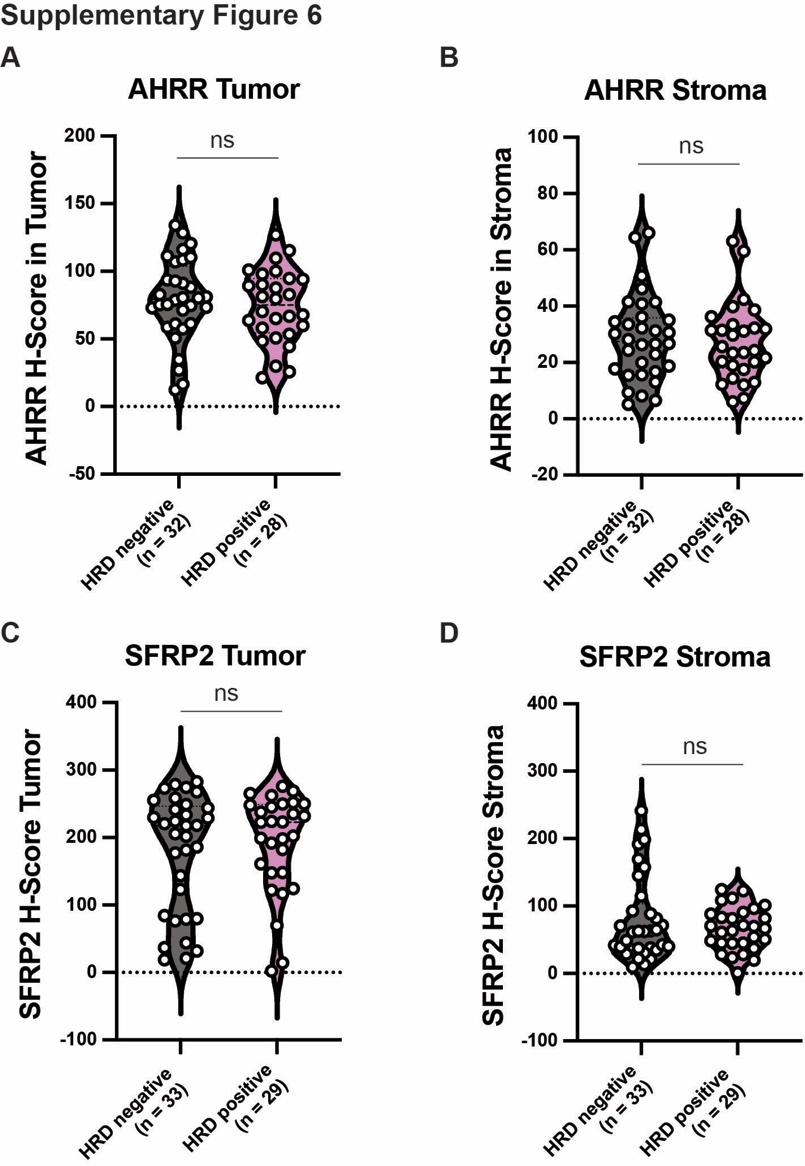
**

**Figure S9: AHRR and SFRP2 expression in HRD positive and negative tumors. A**: AHRR H-Score in tumor cells of HRD positive versus HRD negative HGSOC samples. **B**: AHRR H-Score in stroma cells of HRD positive versus HRD negative HGSOC samples. **C**: SFRP2 H-Score in tumor cells of HRD positive versus HRD negative HGSOC samples. **D**: SFRP2 H-Score in stroma cells of HRD positive versus HRD negative HGSOC samples. P-value: unpaired t-test (A and B) and Mann-Whitney test (C and D). ns = not significant.

**Supplementary Figure S10**


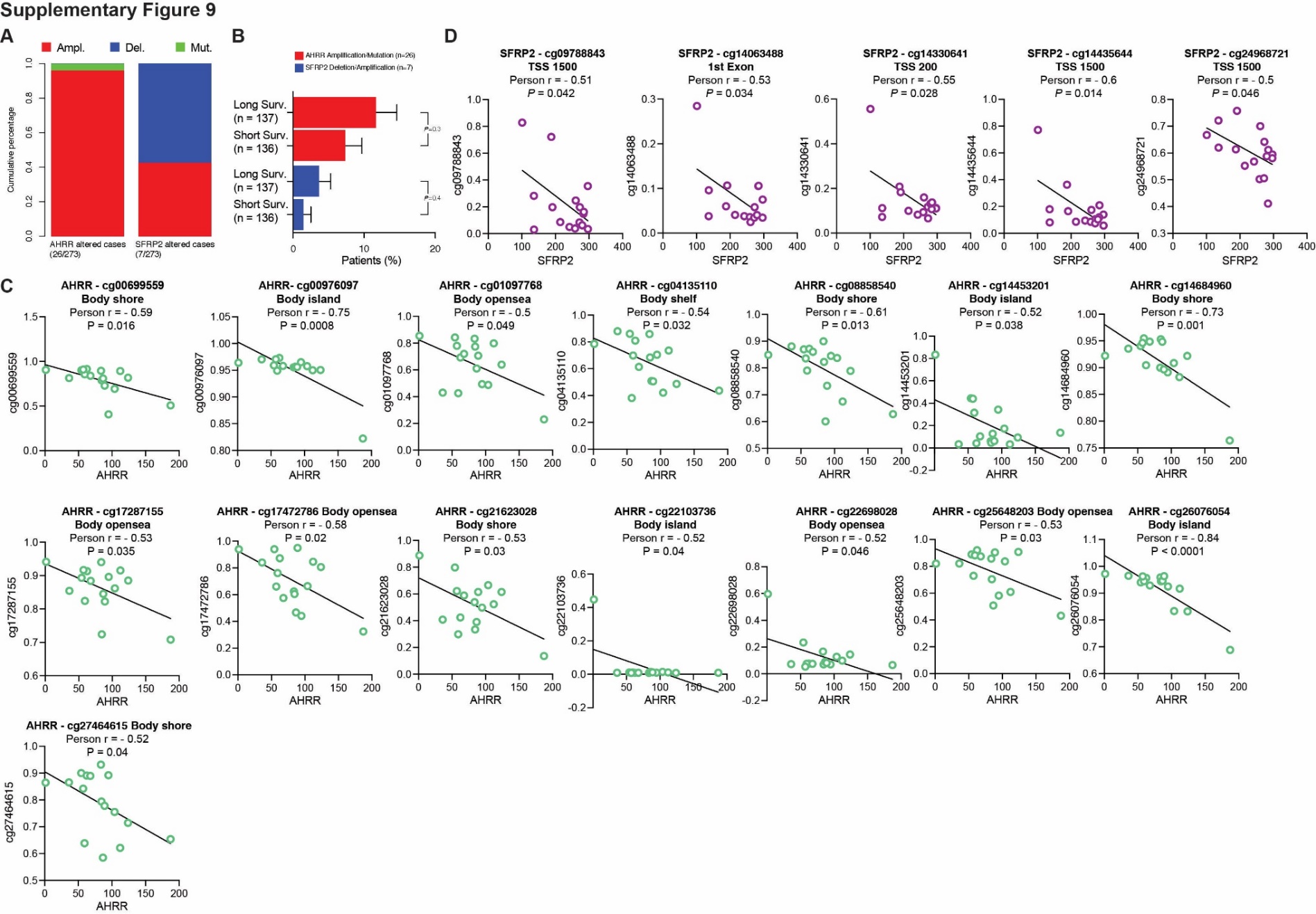


**Figure S10**: **Regulation of AHRR and SFRP2 in ovarian cancer**. **A**. Stacked bar plot representing DNA sequence alterations (mutations, amplifications, and deletions) in *AHRR* and *SFRP2* in 273 ovarian cancer samples. **B**. Comparison of DNA sequence alterations (mutations, amplifications, and deletions) in *AHRR* and *SFRP2* in short- (n=136) versus long-term (n=137) survivors (defined as top/bottom 25% survival length). **C**. CpGs associated with *AHRR* that showed a significant negative correlation between their normalized beta value and the H-score of AHRR protein measured by IHC in a group of 16 patients with HGSOC. **D**. CpGs associated with *SFRP2* that showed a significant negative correlation between their normalized beta value and the H-score of SFRP2 protein measured by IHC in a group of 16 patients with HGSOC. Correlation analysis between parameters was performed using Pearson’s r coefficient.

**Supplementary Figure S11**


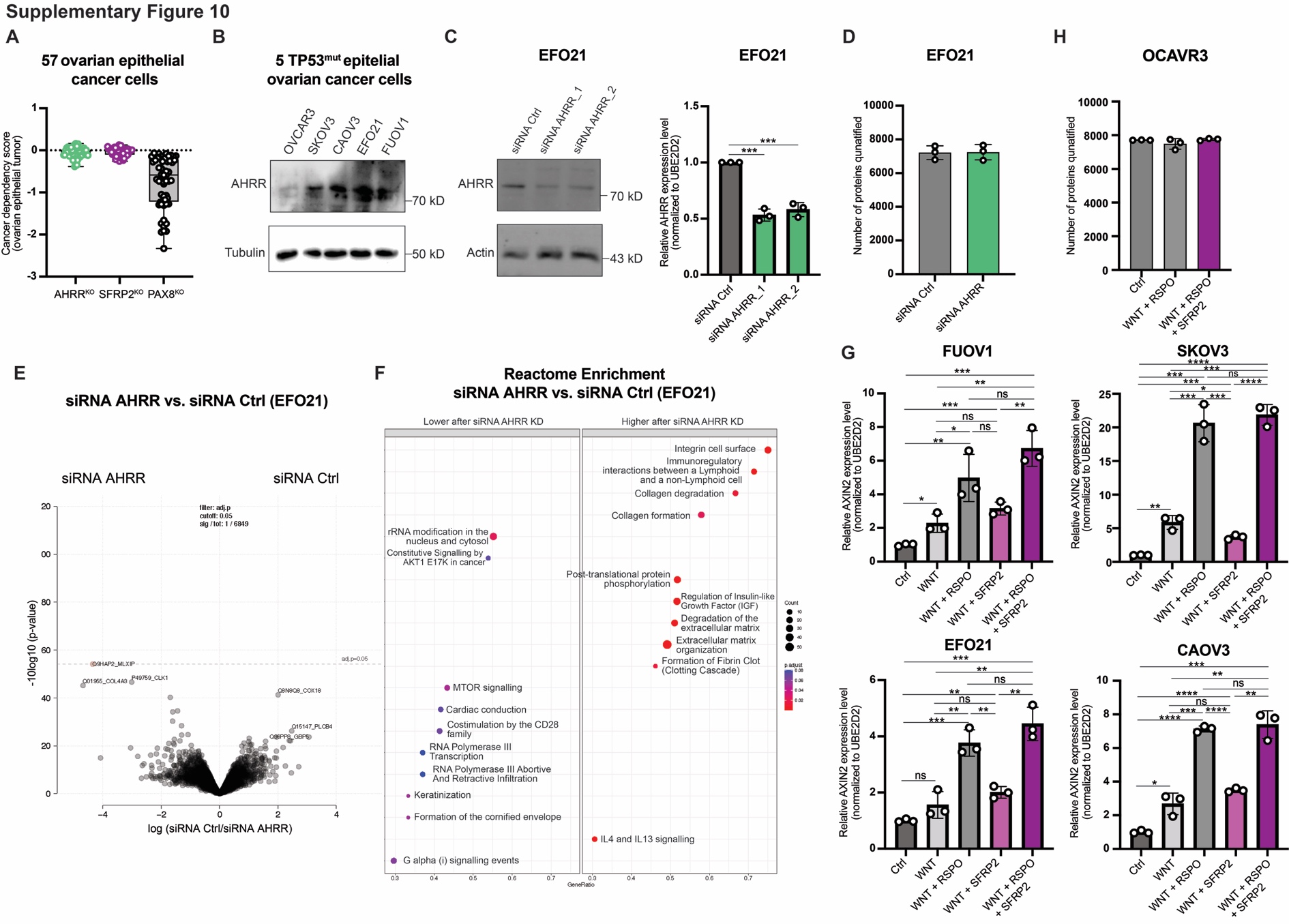


**Figure S11. Functional role of AHRR and SFRP2 in ovarian cancer. A.** Impact of AHRR knock out (AHRR^KO^), SFRP2^KO^ and PAX8^KO^ (positive control) on cell proliferation in 57 epithelial ovarian cancer cell lines (data downloaded from depmap portal). A negative cancer dependency score represents a decrease of the proliferation rate after KO. **B.** AHRR protein expression in five TP53^Mut^ epithelial ovarian cancer cell lines. Tubulin was used as protein normalizer. **C.** Protein (left panel) and mRNA (right panel) level of AHRR analyzed in EFO21 after AHRR knockdown (KD) with siRNAs. Actin was used as protein normalizer. **D.** Number of proteins quantified by MS-based proteomics for the siRNA control and siRNA AHRR groups, three biological replicates per group. The bar plot represents the mean of the three biological replicates, the exact value of each replicate is depicted by the circle, the error bars show standard deviation for each group. **E.** Volcano plot of the pairwise proteomic comparison between the EFO21 siRNA AHRR (KD) and EFO21 siRNA control. Significantly expressed proteins are highlighted in red (moderated two-sided t-test, FDR < 0.05). For both groups, the experiment was done in biological triplicates. **F.** Pathway enrichment analysis (Reactome) based on t-test difference between siRNA control versus siRNA AHRR. Enriched pathways with an FDR < 0.1 are shown. **G.** *AXIN2* mRNA level in FUOV1, SKOV3, EFO21, and CAOV3 cells without treatment (Ctrl), and stimulated with WNT alone, WNT + RSPO, WNT + SFRP2 and WNT + RSPO + SFRP2. **H.** Number of proteins quantified by MS-based proteomics for the siRNA control and siRNA AHRR groups, three biological replicates per group. The bar plot represents the mean of three biological replicates, the exact value of each replicate is depicted by the circle, the error bars show standard deviation for each group. Significance levels were determined using the unpaired t-test (ns = not significant, *p < 0.05, **p < 0.01, *** p < 0.001, **** p < 0.0001).

**Supplementary Tables**

**Supplementary Table S1**. Cohort characteristics: screening, validation, survival, and HRD cohort.

**Supplementary Table S2**. HTG EDgeSeq Oncology Biomarker Panel.

**Supplementary Table S3**. Antibody information.

**Supplementary Table S4**. Settings for digital image analysis in QuPath.

**Supplementary Table S5**. Differentially expressed genes in primary and recurrent HGSOC samples.

**Supplementary Table S6**. Results from the Kaplan-Meier-Plotter analysis (Screening).

**Supplementary Table S7**. Results from the Kaplan-Meier-Plotter analysis for OS and PFS.

**Supplementary Table S8**. Genes with a significant prognostic impact in the Kaplan-Meier-Plotter analysis,

**Supplementary Table S9**. Distribution of protein expression in paired primary and recurrent tumors.

**Supplementary Table S10**. Correlations of protein expression with clinical or histological parameters.

**Supplementary Table S11**. Prognostic impact of the analyzed markers.

**Supplementary Table S12**. Normalized beta values of all CpGs mapping to *AHRR* in 16 patients with HGSOC.

**Supplementary Table S13**. Normalized beta values of all CpGs mapping to *SFRP2* in 16 patients with HGSOC.

**Supplementary Table S14**. All detected proteins and their expression in EFO21 ovarian cancer cell with siRNA AHRR KD (3 replicates) versus siRNA control (3 replicates) as determined by mass spectrometry-based proteomics.

**Supplementary Table S15**. All detected proteins and their expression in OVCAR3 ovarian cancer cell without stimulation (control) (3 replicates), with WNT + RSPO (3 replicates), or with WNT + RSPO + SFRP2 (3 replicates) stimulation as determined by mass spectrometry-based proteomics.

**Supplementary Table S16**. Sample IDs for the in vitro proteomics analysis.

**Supplementary Table S17**. Raw targeted RNA-Sequencing data from 38 HGSOC patients (paired primary and recurrence).

**Supplementary Methods**

**Digital image analysis**

Digital pathological analysis of the IHC-stained TMA slides was performed using QuPath (0.2.2), an open-source software developed for the analysis of complex tissue images ^1^. To identify tumor cores, QuPath’s automated TMA dearrayer was applied to all slides within each project. Color deconvolution and cell detection was applied to identify all cells based on their nuclear staining and with precise characteristics for each biomarker (**Supplementary Tab. S4**). To distinguish cell types and classify cells into tumor cells and cells of the tumor microenvironment (stromal, lymphoid, etc.), a two-way random trees classifier was trained interactively. For this purpose, representative tissue regions were annotated by an experienced user (N.M.) according to the instructions of a senior gyneco-pathologist (E.T.T.). In a final step, individual intensity thresholds were set to subclassify cells as negative, weak, moderate, or strongly positive for the respective biomarker staining based on the mean cytoplasmic or nuclear optical DAB densities (**Supplementary Tab. S4, Fig. 2A**). Quality control of cell detection and classification was performed for each core and adjustments were made if necessary. Detections caused by artifacts were manually excluded. Analysis was performed blinded to the patient survival time.

As a primary outcome of the analysis, a Histoscore (H-score) for tumor and tumor microenvironment was calculated for each tissue sample (3x% strongly stained cells, 2x% moderately stained cells, and 1x% weakly stained cells). This evaluation resulted in a score ranking from 0 (all cells negative) to 300 (all cells strongly positive). As two tumor cores were evaluable per patient, a patient biomarker score was defined as the median of the included cores per patient. If only one TMA core was available for the analysis (due to technical problems), only the H-score of this spot was considered.

**In silico Kaplan-Meier plotter**

We used the Kaplan-Meier plotter (https://kmplot.com/analysis/) ^2^ for a first analysis regarding the prognostic relevance of the differentially expressed genes in the screening cohort. The Kaplan-Meier Plotter uses datasets established before 2012 before the implementation of low- and high-grade serous carcinomas as different entities by the WHO classification in 2014 ^3^. Therefore, the categories low- and high-grade serous carcinoma were not eligible. P53 status was only available for a small number of patients especially in regard to the innovative nature of the genes searched. Because low-grade ovarian carcinomas are far less frequent ^3^ than HGSOC, we opted for the largest possible number of cases and all serous ovarian carcinoma cases (n= 1232) were considered, independent of grade (G), stage (FIGO) or TP53 mutation status. All available datasets were used together, including the TCGA dataset ^4^. Patient groups have been divided in two by an auto-selected cutoff. Correction for multiple testing was applied by the Bonferroni method ^5^.

**Gene term enrichment analysis for differently expressed target genes with prognostic impact**

Gene Ontology (GO; http://geneontology.org) enrichment analysis was performed using the PANTHER classification system ^6^. Gene symbols of the 23 differential expressed genes (DEGs) with a significant prognostic impact (PFS and OS) were uploaded and GO biological processes were analyzed. The HTG EdgeSeq Oncology Biomarker Panel was used as the reference list (**Supplementary Tab. S2**). Fisher’s exact test was applied, and the false discovery rate (FDR) was calculated. Further information on the pathway interactions of each gene were found in the database PathCards (https://pathcards.genecards.org). The top five pathways with the highest relevance score per gene were considered.

**Analysis of genomic aberrations**

We selected samples from the ovarian carcinoma TCGA study ^4^ using the following criteria: serous morphology, G2/G3 grading, available survival data, and successful DNA profiling. Next, based on the days to death from diagnosis, we divided the cohort into short-term survivors (top 25% shortest survival time) and long-term survivors (top 25% longest survival time). We ended up with a cohort of 273 tumors, 136 short survivors and 137 long survivors. We accessed openly available mutation and copy number data from cBioPortal ^7^ and analyzed gene alteration frequencies for *AHRR* and *SFRP2*.

**In silico cell proliferation analysis**

Chronos dependency score data for AHRR, SFRP2 and PAX8 were obtained from the depmap portal for all ovarian epithelial cancer models (downloaded from https://depmap.org/portal/download/all/). PAX8 as a lineage-specific survival gene was perceived as a positive control. GraphPad Prism 9 was used to plot the box-plots of each gene across 57 epithelial ovarian cancer models on a gene-by-gene basis.

**Cell lines**

OVCAR3 (established from the malignant ascites), SKOV3 (isolated from the ovary), CAOV3 (established from the ovary) were obtained from ATCC, and EFO21 (established from the malignant ascitic fluid), and FUOV1 (established post-hysterectomy) were obtained from DSMZ. The cells were cultured in in DMEM (Gibco #21885-025), all supplemented with 10% fetal bovine serum (FBS), no added antibiotics, at 37°C with 5% CO2 and 95% humidity. Prior to the study, the cytogenetic analysis and cell authentication of the cells was performed at the DNA-Fingerprinting Facility at Charité Berlin using short tandem repeat DNA. All cell lines were tested for mycoplasma contamination using PCR mycoplasma kit (Biontex #M030/050).

**WNT signaling activation and SFRP2 stimulation**

OVCAR3, SKOV3, CAOV3, EFO21, and FUOV1 cells were cultured in DMEM plus FBS. For WNT target activation assays, the cells were seeded into 6-well plates and 24 hours later were cultured using the following conditions: Control (no growth factors added); WNT (using conditioned DMEM from WNT3-transgenic L-cells containing the equivalent of 100 ng/ml WNT) ^8^; WNT + RSPO1 (additionally adding conditioned medium from R-Spondin1-Fc transgenic 293 cells) ^8^; WNT + SFRP2 (additionally adding 0.1 µg/ml rhsFRP-2 (R&D No 6838-FR)); and WNT + RSPO1 + SFRP2. After 48 hours incubation the cells were harvested for RT-qPCR. Cells were cultured under the same conditions for MS analysis.

**RNA Interference**

Two siRNAs against two different AHRR exons (Catalog# AM16708 siRNA ID 112109, and siRNA ID 112108) or negative control siRNA (Catalog# 4390843) were purchased from Invitrogen™. Cells were seeded into 6-well plates. When the cells reached 60% confluence, they were transfected with 25 nM of the corresponding siRNA by Oligofectamine (Invitrogen™) according to the manufacturer’s protocol. RNA and proteins were collected at 48 hours after transfection. Quantitative real time PCR (qRT-PCR) and Western blot were used to check the efficiency of knock-down.

**RT-qPCR**

For total RNA extraction, cells were washed twice with cold PBS, lysed and RNA was subsequently isolated using RNeasy Kit and RNeasy Micro Kit (QIAGEN) according to the manufacturer’s specification. RNA (1 μg) was reverse- transcribed using Transcriptor High Fidelity cDNA Synthesis Kit (Roche) using both random hexamer and oligo(dT) primer. The qPCR was performed in triplicates with 1 μl of 1:20 to 1:100 diluted cDNA using 14 μl of GoTaq Mastermix (Promega) and 1 μl primer mix (333 nM each) (*UBE2D2* fw CAGTAATGGCAGCATTTGTCTTGA, rv TCATCTGGATTGGGATCACACA; *AHRR* fw CGTTCCCGCCTGACATCAT, rv TCTGCTCCTGCACGACTTG; *AXIN2* fw AGAGCAGCTCAG CAAAAAGG, rv CATCCTCCCAGATCTCCTCA and analyzed with StepOne Real-Time PCR System (Applied Biosystems). The relative gene expression levels were calculated using the 2^-ΔΔCt^ method. *UBE2D2* was used as housekeeping gene for normalization. The absolute RNA expression of the housekeeping genes showed a minimal and nonstatistical difference between the groups we compared.

**Western blot**

The cells were washed twice in cold PBS and subsequently lysed in M-PER Mammalian Protein Extraction Reagent (ThermoFischer) containing PhosSTOP™ and cOmplete™ protease inhibitor cocktail (Roche) for 10-30 min on ice. The lysate was sonicated twice for 5 min in 30 s intervals (Diagenode Bioruptor) before debris was removed by centrifugation at 13,000 rpm for 15 min at 4°C. Protein concentrations were determined using Pierce BCA Protein Assay Kit (ThermoFischer) and equal amounts of proteins (20 μg) were separated on SDS-PAGE (10%) and transferred onto PVDF (Immobilon, Merck, Millipore Ltd.). The membrane was blocked with 5% non-fat dry milk in TBS-T (25 mM Tris, 150 mM NaCl, 2 mM KCl, pH 7.4 containing 0.1% Tween-20) for 1 h at room temperature and thereafter incubated overnight at 4°C with the primary antibodies diluted in 5% non-fat dry milk in TBS-T. Primary antibodies: AHRR (1:3000, Abcam, Ref. No ab108518); Actin Primary Antibody hFAB™ Rhodamine (BioRad, 12004164) 1:2000; and Tubulin (1:1000, CellSignaling, #2146). A washing series with TBS-T followed, and membranes were subsequently probed with the secondary antibody conjugated to horseradish peroxidase for 1 h at room temperature: anti-rabbit (CellSignaling, #7074), 1:2000. The membranes were washed again as described above, and chemiluminescence was detected using GE Healthcare ECL (Amersham ECL).

**Sample preparation for mass spectrometry (MS) based proteomics**

Cell lysis from frozen cell pellets (approx. 1E6 cells, **Supplementary Tab. S16**) was performed in lysis buffer (4% SDS, 25 mM ammonium bicarbonate, pH 8.0) at 95 °C for 10 min followed by 15 min sonication (Bioruptor, Diagenode) at 4 °C. Proteins in the lysate were reduced with 10 mM DTT for 30 min and alkylated with 55 mM 2-chloroacetamide for an additional 30 min at room temperature in darkness. Proteins were then purified by acetone precipitation. Briefly, acetone (-20 °C) was added to a final concentration of 80% v/v and the proteins were precipitated overnight at -20 °C. After centrifugation (15 min, 4 °C, 16,000 g), the detergent-containing supernatant was removed and the protein pellet was washed with 80% acetone (-20 °C). Protein pellets were then resolved in 100 μl 8M urea, sonicated for 15 min at 4 °C. Protein concentration was measured by BCA assay (Thermo Fisher Scientific), and 20 μg of each sample was used for digestion in 50 μl 8M urea (prepared in 25 mM Hepes pH 8.0). First, 200 ng of LysC (Promega, cat.no VA1170) was added and the samples digested for 3h at room temperature under agitation. After diluting the reaction mixture fourfold with 150 μl of 50 mM ammonium bicarbonate, 200 ng of trypsin (Promega, cat.no V5117) was added and incubated overnight at room temperature. The next day, digestion was stopped by adding 1% trifluoroacetic acid (TFA). Peptides were desalted on SDB-RPS StageTips (3M Empore), eluted into a 96 well plate (Thermo Fisher Scientific, cat.no. AB1300), vacuum concentrated and stored at -20 °C until LC-MS analysis. For LC-MS analysis, 200 ng of each sample was injected in MS loading buffer (3% acetonitrile in 0.1% formic acid).

**MS raw file analysis and proteomics statistical analysis**

We used DIA-NN (version 1.8.1) for dia-PASEF raw file analysis with library-free approach. We used human FASTA file downloaded from Uniprot (2022 release, UP000005640_9606, downloaded on April 8th 2022) and frequently found contaminants (human + universal contaminants) ^9^. Deep learning-based spectra, RTs and IMs prediction were enabled for the appropriate mass range of 300-1200 m/z. N-terminal M excision was enabled and cysteine carbamidomethylation was enabled as a fixed modification. A maximum of 2 missed cleavages was allowed, and the precursor charge set to 2 - 4. MS1 and MS2 accuracies were set to 15.0, scan windows to 0 (assignment by DIA-NN), isotopologues were enabled, MBR activated, heuristic protein inference and no shared spectra. Proteins were inferred from genes, neural network classifier was set to single-pass mode, quantification strategy as ‘Robust LC (high precision)’. Cross-run normalization was set to ‘RT-dependent’, library generation as ‘smart profiling’, speed and Ram usage as ‘optimal results’. The generated library consisted of 155642 precursors, 133804 elution groups, 111941 protein groups and 11128 protein isoforms. The pg.matrix.tsv output file containing the relative protein level information was used for downstream data analysis.

Proteomics data analysis was performed with Perseus (version 1.6.15.0, ^10^ and in R (Protigy version 0.9.1.4, <https://github.com/broadinstitute/protigy>). Data were filtered to keep only proteins with minimum number of valid values of 3 in at least one group. Missing values were imputed based on a normal distribution (width = 0.3, downshift = 1.8) before statistical testing. For differential expression analysis, we used a moderated two-sample T-test with adjusted p-value < 0.05. Pathway enrichment analysis was done in clusterProfiler ^11^, with an FDR < 0.1 and a minimum category size of 10.

**References**

1. Bankhead P, Loughrey MB, Fernandez JA, Dombrowski Y, McArt DG, Dunne PD *et al.* QuPath: Open source software for digital pathology image analysis. *Sci Rep* 2017; **7**(1): 16878.

2. Gyorffy B, Lanczky A, Szallasi Z. Implementing an online tool for genome-wide validation of survival-associated biomarkers in ovarian-cancer using microarray data from 1287 patients. *Endocr Relat Cancer* 2012; **19**(2): 197-208.

3. Seidman JD, Bell DA, Crum CP, Gilks CB, Kurman RJ, Levine DA *et al.* High-grade serous carcinoma. In: Kurman RJ (ed.) *WHO Classification of Tumours of Female Reproductive Organs* International Agency for Research on Cancer: Lyon, 2014, pp 22-24.

4. Cancer Genome Atlas Research N. Integrated genomic analyses of ovarian carcinoma. *Nature* 2011; **474**(7353): 609-615.

5. Andrade C. Multiple Testing and Protection Against a Type 1 (False Positive) Error Using the Bonferroni and Hochberg Corrections. *Indian J Psychol Med* 2019; **41**(1): 99-100.

6. Mi H, Muruganujan A, Casagrande JT, Thomas PD. Large-scale gene function analysis with the PANTHER classification system. *Nat Protoc* 2013; **8**(8): 1551-1566.

7. Cerami E, Gao J, Dogrusoz U, Gross BE, Sumer SO, Aksoy BA *et al.* The cBio cancer genomics portal: an open platform for exploring multidimensional cancer genomics data. *Cancer Discov* 2012; **2**(5): 401-404.

8. Brandt R, Sell T, Luthen M, Uhlitz F, Klinger B, Riemer P *et al.* Cell type-dependent differential activation of ERK by oncogenic KRAS in colon cancer and intestinal epithelium. *Nat Commun* 2019; **10**(1): 2919.

9. Frankenfield AM, Ni J, Ahmed M, Hao L. Protein Contaminants Matter: Building Universal Protein Contaminant Libraries for DDA and DIA Proteomics. *J Proteome Res* 2022; **21**(9): 2104-2113.

10. Tyanova S, Temu T, Sinitcyn P, Carlson A, Hein MY, Geiger T *et al.* The Perseus computational platform for comprehensive analysis of (prote)omics data. *Nat Methods* 2016; **13**(9): 731-740.

11. Yu G, Wang LG, Han Y, He QY. clusterProfiler: an R package for comparing biological themes among gene clusters. *OMICS* 2012; **16**(5): 284-287.
